# Increasing Health Literacy on ADHD: A Cross-Disciplinary Integrative Review Examining the Impact of ADHD on Brain Maturation, Composition and Function and Cognitive Processes Across the Life Course

**DOI:** 10.1007/s10578-025-01815-5

**Published:** 2025-02-26

**Authors:** Louise E. Brown, Mary Tallon, Mark A. Bellgrove, Daniel Rudaizky, Garth Kendall, Mark Boyes, Bronwyn Myers

**Affiliations:** 1 Faculty of Health Sciences, enAble Institute, Curtin University, Perth, WA 6845 Australia; 2 Faculty of Health Sciences, School of Nursing, Curtin University, Perth, WA 6845 Australia; 3 School of Psychological Sciences, Turner Institute for Brain and Mental Health, Monash University, Clayton, VIC 3800 Australia; 4 Faculty of Health Sciences, School of Population Health, Curtin University, Perth, WA 6845 Australia

**Keywords:** ADHD, Health literacy, Child development, Neuropsychological development, Cognition

## Abstract

There is a significant need to improve ADHD health literacy. This cross-disciplinary integrative review was conducted to synthesise the evidence on the impact ADHD has on brain maturation, composition and function as well as cognitive processes, across the life course. Although results are highly heterogenous, ADHD appears to be associated with (1) a significant delay in cortical maturation and differences in neuroanatomy that do not appear to fully resolve in adulthood, (2) atypical brain function, and (3) atypical cognitive processes. The cognitive processes implicated include working memory, inhibitory control, cognitive flexibility, alerting attention, reward processing, long-term memory, reaction time, time perception and estimation, planning, and complex decision making/problem-solving. We aim to use this data to develop a ‘framework/checklist” that parents, adults and clinicians can use to identify the possible mechanisms that may be contributing to an individual with ADHD’s challenges. This information can also be used to inform the content of ADHD education programs to ensure participants receive empirically-determine information from high quality review studies and meta-analysis that accurately reflects the rigor and limitations of study findings.

**Supplementary Information:**

The online version contains supplementary material available at https://doi.org/10.1007/s10578-025-01815-5.

## Introduction

Attention deficit hyperactivity disorder (ADHD) is a complex neurodevelopmental condition (American Psychiatric Association, [1]) that remains relatively misunderstood by health care practitioners, teachers, parents, and individuals with the condition [2]. Characterised by persistent inattentive and/or hyperactive-impulsive symptoms that are inconsistent with a person’s developmental level (American Psychiatric Association, [1]), ADHD affects the ability to exert age-appropriate self-control [3]. Across the life course, this highly genetic condition [4, 6] significantly impacts a person’s neuropsychological development, everyday functioning, mental and physical health, quality of life, and life expectancy [7, 9].

Evidence-based clinical practice guideline for ADHD, such as the Australian and UK guidelines [10], National Institute for Health and Care Excellence [NICE], [11]), recommend the provision of ADHD-related education and training to healthcare providers and teachers, and psychoeducation to people with ADHD and their families. These recommendations are echoed by people with lived experience of ADHD and their families who report low rates of ADHD-related literacy and an unmet need for ADHD education [2]. Providing evidence-based education on ADHD, however, is challenging. Not only are research findings difficult to access, consensus on the pathophysiology and neuropsychology underlying ADHD has not been achieved. Scientists are continuing to learn about the brain’s functional architecture [12], and what is ‘known’, is not necessarily absolute. It reflects (1) converging evidence from diffusion tensor imaging (DTI), structural and functional magnetic resonance imaging (MRI; fMRI, respectively), positron emission tomography (PET), single-photon emission computed tomography (SPECT), neurophysiology (electroencephalography, EEG), post-stroke lesion studies, and post-mortem anatomic dissection, and (2) scientists’ interpretations of how brain structure and connectivity translates into cognitive, behavioural, social and emotional outcomes [12]. Furthermore, the interpretation of study findings is complicated by factors such as the brain’s structural and functional complexity, and clinical (participant age, comorbidity) and study (specialty, methodological, data interpretation) heterogeneity [12, 13]. Other factors that have likely impeded the advancement towards consensus include the assumption that ADHD-associated cognitive challenges underpin ADHD symptoms rather than coexist and make independent contributions to ADHD presentation, and ADHD-related heterogeneity [14]. People with ADHD can present with a range of situationally variable symptoms that tend to change across the life course, as well as different neurocognitive profiles and developmental trajectories [15].

Regardless of these challenges, the need for education that improves healthcare practitioner, teacher, parent and consumer ADHD-related health literacy remains [2]. The aim of this cross-disciplinary integrative review is to synthesise the evidence on the impact ADHD has on brain maturation, composition and function as well as cognitive processes, across the life course to develop a single source of empirical evidence on the biologically-based, innate person characteristics that may influence a child or adult with ADHD’s developmental trajectory and functional capacity, as well as their mental wellbeing and other socio-emotional outcomes. As per Bronfenbrenner’s [16] Bioecological model of human development, an individual’s biologically-based person characteristics interact with the broader environment, and along with the subjective experiences they elicit in them, influence their actualisation of potential and their socio-emotional outcomes across the life course. This information is therefore needed to provide insights into the possible neurobiological and cognitive mechanisms underlying ADHD-related functional, mental health and socio-emotional outcomes and to support self-awareness, acceptance, recovery and personal empowerment of persons with ADHD.

By making this scientific knowledge more accessible we hope that it will be used to inform the content of evidence-based ADHD education programs. We acknowledge that the way in which the information is presented, however, will need to be adjusted to ensure that it is comprehensible and meaningful to recipients. We will be using the findings of the review to develop a ‘framework/checklist” that parents, adults and clinicians can use to identify the possible mechanisms that may be contributing to an individual with ADHD’s challenges. In line with the complexity associated with ADHD (including the heterogeneity and impact of situational variability), this framework/checklist will serve as a tool for identifying possible ADHD-related factors that ‘may’ impact on the achievement of a desired end state, and to identify the scaffolding that may be required to facilitate recovery and personal empowerment.

## Method

This integrative review was conducted according to the stages outlined by Whittemore & Knafl [17]: problem identification, literature search, data evaluation, data analysis, and presentation of synthesised findings. Integrative methodology was chosen as it enables researchers to examine a phenomenon more broadly; systematically assimilate data from a diverse range of research specialities, methodologies and perspectives in order to advance understanding of complex concepts and phenomena; and draw evidence-based, holistic conclusions and new perspectives that promote better understanding of a topic and can be used to inform clinical practice [17–19].

## Search Strategy

A strategy for searching ‘Medline’, ‘American Psychology Association (APA) PsycArticles’ and ‘Cumulated Index to Nursing and Allied Health Literature (CINAHL)’ databases was developed with the assistance of a Curtin University Librarian. Broad search terms were chosen that would enable us to capture the breadth of international papers on the topic. For the ‘Medline’ search, the following MESH heading was used: ‘Attention Deficit Disorder with Hyperactivity/’. For the APA PsycArticles’ and ‘CINAHL’ searches, as well as a ‘Medline’ search that was limited to 2023, the following keywords were used: ‘Attention Deficit Disorder with Hyperactivity/’, ‘Attention Deficit Hyperactivity Disorder’, ‘Attention Deficit-Hyperactivity Disorder’, ‘Deficit-Hyperactivity Disorder, Attention or Disorder’, ‘Attention Deficit-Hyperactivity or Hyperkinetic Syndrome’, ‘Hyperkinetic or Attention Deficit Disorder’, ‘Attention or Brain Dysfunction, Minimal Brain’, ‘Minimal Brain Dysfunction’, ‘Minimal cerebral dysfunction’ or ‘ADHD’. All searches were limited to review studies that synthesised findings from primary empirical studies (i.e., meta-analysis; systematic, structured, integrative and scoping reviews), that were written in English and published between January 1998 and July 2023. This timeline was chosen because the first review papers examining the findings of magnetic resonance imaging (MRI) studies assessing the brain structure of children with ADHD were published in 1998. The search strategy ensured that a manageable number of studies would be captured from a wide range of specialties (i.e., neuroscience, medical, psychology and nursing) employing different methodologies (experimental and non-experimental/observational) and ontological (i.e., biomedical, social constructivist, and critical realist) and epistemological paradigms (i.e., positivism, interpretivism, critical theory and pragmatism).

LEB conducted a search of all databases using this strategy on 4th December 2023. The reference lists of studies that met the study inclusion/exclusion criteria were also searched by LEB to identify any relevant reviews not captured by the database searches. Because none of the six pertinent longitudinal case–control neuroimaging studies captured during these searches, which were deemed important to include as they provide insight into the age-related and brain-related differences associated with ADHD, were included in any reviews that met the inclusion/exclusion criteria, or in the case of Shaw (2013; 2012) and Proal (2011) included in their entirety, the decision was made to include these studies in this review. An additional purposeful search was then carried out on the 20th December 2023 to identify any other relevant but not captured studies. During this search, two large cross-sectional ENIGMA neuroimaging studies examining anatomical brain structure with greater than 2,000 participants, were identified [20, 21]. After confirming these papers were not included in any of the reviews, the decision was made to also include these papers.

### Inclusion Criteria

Review studies were included if they reported on the influence of ADHD on brain maturation, composition (anatomy and morphology) and function, or cognitive processes. Reviews examining other conditions along with ADHD (e.g., autism) were included so long as they compared participants with ADHD against neurotypical developing peers (See Supplementary information, Appendix A for the full inclusion/exclusion criteria). As noted above, the decision was made to also include six longitudinal case–control and two large cross-sectional neuroimaging studies to ensure their findings were reflected in the integrative review findings.

### Procedure

The titles and abstracts of identified papers were screened by LEB. Those that met the eligibility criteria proceeded to full text review by LEB. A random 20% of these full texts were independently screened by MT. Discrepancies were discussed and resolved. Critical appraisal was then conducted by LEB, with MT independently appraising the quality of a randomly selected 20% as well as providing input as requested. An adapted version of the JBI Critical Appraisal Checklist for Systematic Reviews and Research Syntheses [22] was used to screen all review studies. The longitudinal case–control and the large cross-sectional studies were screened using an adapted version of the AXIS Critical Appraisal of Cross-Sectional Studies [23] (Both tools are available in the Supplementary Information, Appendix B). Following discussion and the resolution of discrepancies, studies were allocated a quality score ranging from zero to seven. This score was converted to a high, moderate, low or very low-quality ranking, using the following criteria: 0—2 = high, 3 or 4 = moderate, 5 or 6 = low, and 7 = very low.

### Data Extraction and Synthesis

For each full-text paper, LEB extracted information on: authors; year of publication and country; methods; number and type of studies captured (psychophysiological and brain-imaging, or neuropsychological); sample size and characteristics (age range [children, adolescents, adults], co-existing conditions); summary of results in comparison to neurotypical peers; and reported heterogeneity and risk of publication bias. Twenty percent of this data was independently cross-referenced by DR for accuracy. Discrepancies were discussed and resolved. Table 1.

**Table 1:** Included studies

First author	Year	Method	Studies /type	Sample	Mean age/years	Quality	Results in comparison to neurotypical peers
Alderson	(2013)	SR MA	38 N	ADHD n = 1338 Controls n = 1393	Adults	Mod	Adults with ADHD displayed atypical phonological and visuospatial working memory (moderate effect size), indicating working memory problems persist beyond childhood. Several task-moderating variables explained significant effect size variability (heterogeneity) among studies. Risk of publication bias was non-significant
Alderson	(2007)	SR MA	25 N	ADHD n = 808 Controls n = 695	Children	Mod	Children with ADHD displayed significantly slower mean reaction time, greater reaction time variability, and slower stop-signal reaction time (moderate effect size). Findings pertain to executive-motor inhibition. Several subject and task variables served as significant moderators for children’s mean reaction time (heterogeneity). Risk of publication bias not assessed
Aoki	2018	SR MA	27 P	ADHD n = 871 Controls n = 846	Children Adolescents Adults	Mod	TBSS: children and adults with ADHD display significantly lower fractional anisotropy values in four clusters (two in the corpus callosum, one in R inferior fronto-occipital fasciculus, and one in L inferior longitudinal fasciculus). WBVBA: elevated fractional anisotropy in three clusters (L mid-cingulate, extending to the corpus callosum, anterior corpus callosum, and L inferior fronto-occipital fasciculus). Results from different methodology did not converge and many studies did not examine potential group differences in head motion. In studies that did, most reported no significant results. Risk of publication bias not assessed
Aoki	2013	MA	16 P	ADHD n = 270 Controls n = 235	Children Adolescents Adults	High	Childhood ADHD is associated with significantly higher-than-normal N-acetylaspartate, a marker of neuronal activity, in the medial prefrontal cortex. No significant difference was found in adults with ADHD and meta-regression revealed a significant correlation between advanced age and normal levels of N-acetylaspartate in the ADHD cohort. Results did not reveal publication bias and heterogeneity was negligible
Arns	2013	MA	9 P	ADHD n = 1253 Controls n = 517	Children Adolescents	Low	Children with ADHD displayed elevated theta/beta ratio (moderate effect size). Results were marred by significant heterogeneity and are therefore considered misleading. Risk of publication bias not assessed
Arora	2020	MA	9 N	ADHD n = 491 Controls n = 402	Children	Low	Children with ADHD displayed atypical alerting and executive attention networks. Non-zero heterogeneity. Risk of publication bias not accessed
Balogh	2016	MA	15 P	ADHD n = 1053 Controls n = 614	Children Adolescents Adults	Mod	Children and adults with ADHD displayed significantly diminished post error slowing after committing an error (medium effect size), regardless of gender. They also tended to sustain or even increase the speed of their reactions post error. Risk of publication was non-significant
Bellato	2020	SR	55 P	ADHD n = > 1400 Controls n = > 1400	Children Adolescents Adults	Low	Children and adults with ADHD tended to display atypical autonomic nervous system arousal, more often in the direction of hypo-arousal than hyper-arousal, particularly at rest and during tasks requiring response regulation and sustained attention. Half the reported findings were null. Publication bias not assessed. It is too early to say whether atypical autonomic nervous system dysfunction is related to ADHD as a continuous trait measure
Boedhoe	2020	C/S ENIGMA	-	ADHD n = 2271 ASD n = 1777 OCD n = 2323 Controls n = 5827	Children Adolescents Adults	High	Children and adolescents with ADHD displayed reduced intercranial volume, reduced frontal surface area and striatal volume in comparison (small to moderate effect size). None of the subcortical volumes differed significantly among adult patient groups
Boon	2020	SR	174 P N	n = > 8000	Children Adolescents	Low	Children and adolescents with ADHD displayed significant differences in neural anatomy, connectivity and innovation, and cognitive functioning. Publication bias not assessed
Boonstra	2005	MA	13 N	ADHD n = 662 Controls n = 417	Adults	Low	Adults with ADHD displayed atypical verbal fluency, inhibition, and set-shifting, as well as atypical reaction time (moderate effect size). Heterogeneity and risk of publication bias not accessed
Chamorro	2022	MA	31 P	n = 1567	Adolescents Adults	High	Individuals with ADHD commit more ocular inhibition errors in the form of anticipatory rapid eye movement responses during memory guided saccade task waiting periods (moderate to large). Risk of publication bias corrected during meta-analysis. Results suggests that the capacity to withhold a response on presentation of a relevant stimulus is impaired in adolescents and adults with ADHD. Due to significant heterogeneity results should be interpreted with caution
Chen	2023	MA	16 N	ADHD n = 2928 Controls n = 2775	Children Adolescents Adults	High	Children and adults with ADHD consistently displayed significantly smaller amygdala surface area (particularly in the L hemisphere), without a significant difference in volume. The effects of the scanner types, segmentation methods, and the investigated continuous variables (age, IQ, and the male percentage) on ADHD-associated anatomical alterations of the amygdala size were nonsignificant. Risk of publication bias was non-significant
Chen	2016	SR MA	10 P	ADHD n = 470 Controls n = 479	Children Adolescents Adults	High	Individuals with ADHD displayed significant fractional anisotropy reductions in the commissural fibres that connect bilateral hemispheres; the association fibres that link occipital, frontal and temporal regions; and projection fibres that pass through the corona radiata and cerebellum. The most evident and consistent white matter differences were located in the splenium of the corpus callosum extending to the R cingulum, the R sagittal stratum and L tapetum. The projection fibres that pass through the corona radiata and cerebellum may also be affected, although sensitivity analysis failed to retain significance. Significant between-group heterogeneity was detected in the splenium of the corpus callosum and the R sagittal stratum. Results were free from publication bias and indicate that the interruptions in the white matter tracts are related to interhemispheric communication, posterior brain circuitries and the limbic system
Cheng	2016	SR MA	6 P	ADHD n = 73 Controls n = 75	Children Adolescents	High	Children and adolescents with ADHD demonstrated significantly smaller mismatched negativity amplitudes, indicating less vigilance to auditory change, reduced involuntary attention switching, and poor auditory sensory memory—a component of working memory (small affect size). No statistical evidence was found for publication bias or heterogeneity
Connaughton	2022	SR	46 P	ADHD n = 1589 Controls n = 1410	Children Adolescents	Mod	Children and adolescents with ADHD tended to display widespread atypical white matter microstructure in both discrete white matter tracts and neural networks (mostly decreased connectivity but also increased in some areas). The most prominent differences were found in the fronto-striatal tracts, corpus callosum, superior longitudinal fasciculus, cingulum bundle, thalamic radiations, internal capsule and corona radiata. Many of the affected neural networks are associated with key neuropsychological functions that are atypical in ADHD. Heterogeneity in the literature may stem from a variety of methodological limitations. Risk of publication bias not assessed
Cortese	2021	SR MA	30 P	ADHD n = 1094 Controls n = 884	Children Adolescents Adults	Mod	Although studies report ADHD-related hyperconnectivity and hyperconnectivity, no spatial convergence was achieved. This may be due to heterogeneity in study participants, methodology, experimental procedures, and analytic flexibility as well as in ADHD pathophysiology. Post hoc meta-analysis showed ADHD is associated with consistently dysregulated L superior temporal gyrus, which is involved in auditory processing and social cognition. Risk of publication bias not assessed
Cortese	2016	MA	24 P	ADHD n = 422 Controls n = 433	Adults	Low	Adults with ADHD displayed hypoactivation in the L putamen, L inferior frontal gyrus (pars opercularis), L temporal pole, and R caudate. The L inferior gyrus and R caudate mapped to executive function tasks. When focusing on studies including participants free of comorbidity, only one region (L putamen) was found to be consistently hypoactivated. Risk of publication bias not assessed
Cortese	2012	SR MA	55 P	ADHD n = ≈ 741 Controls n = ≈ 801	Children Adolescents Adults	High	Children with ADHD displayed significant (1) hypoactivation in the frontal, R parietal and R temporal regions, putamen bilaterally and in the systems that govern executive function (frontoparietal network) and attention (ventral attentional network), and (2) hyperactivation in the R angular gyrus, middle occipital gyrus, posterior cingulate cortex, and midcingulate cortex and in the default, ventral attention, and somatomotor networks. Stimulant-naïve children displayed hypoactivation in the R superior temporal gyrus, bilateral putamen and R thalamus. Adults with ADHD displayed significant (1) hypoactivation in the R central sulcus, precentral gyrus, and middle frontal gyrus, and frontoparietal system; and (2) hyperactivation in a region with a peak in the R angular and middle occipital gyri, and in the visual, dorsal attention, and default networks. ADHD-related dysfunction largely reflected task features and was detected even in the absence of comorbid mental disorders or a history of stimulant treatment. Risk of publication bias not assessed
Cortese	2006	SR MA	13 N	ADHD n = 280 Controls n = 228	Children Adolescents	Mod	Children with ADHD display significantly reduced mean sleep latency (large effect size), fall asleep more often during multiple sleep latency testing (large effect size), and may display excessive daytime sleepiness which impacts alertness. Children with ADHD display significantly more movement during sleep, a significantly higher apnoea-hypopnea index and sleep-disordered breathing. Risk of publication bias not assessed
Cui	2023	MA	22 P	ADHD n = 1158 Controls n = 1204	Children Adolescents Adults	High	Individuals with ADHD displayed reliable and robust age-related significantly reduced fractional anisotropy in the splenium of the corpus collosum. The adult ADHD subgroup also displayed reduced fractional anisotropy in the body of the corpus collosum, indicating white matter tract integrity reduces with increasing age. No clear evidence of between-study heterogeneity or publication bias identified
Dan	2020	IR	9 P N	ADHD n = 313 Controls n = 327	Children Adolescents	Very Low	Adolescents with ADHD display no differences in reaction time, reaction time variance and cognition accuracy when recognising emotional facial expressions, however, brain activity and temporal evolution differences are present when neural responses are recorded using f-MRI or event related potentials. Risk of publication bias not assessed
Dekkers	2016	SR MRA	37 N	ADHD n = 1175 Controls n = 1222	Children Adolescents Adults	High	ADHD is related to significantly increased risky decision making on gambling tasks in laboratory settings (small to medium effect size). Larger effect sizes were associated with co-morbid Disruptive Behaviour Disorder. Age did not moderate outcomes. No evidence of publication bias
Dickstein	2006	MA	16 P	ADHD n = 184 Controls n = 187	Children Adolescents Adults	Low	Across studies, individuals with ADHD displayed significant patterns of frontal hypoactivity within the anterior cingulate, dorsolateral prefrontal, inferior prefrontal, and orbitofrontal cortices, as well as related regions, such as portions of the basal ganglia and parietal cortices. When focusing on studies of response inhibition alone, a more limited set of group differences were observed, including inferior prefrontal cortex, medial wall regions (including anterior cingulate cortex) and the precentral gyrus. In contrast, analyses focusing on studies of constructs other than response inhibition revealed a more extensive pattern of hypofunction in patients with ADHD than those of response inhibition. Risk of publication bias not assessed
Doidge	2021	MA	28 N	ADHD n = 1055 Controls n = 3485	Children Adolescents Adults	Mod	During temporal discounting and delay of gratification tasks, females with ADHD chose smaller immediate rewards over larger delayed rewards more often than males with ADHD. In contrast, males with ADHD were more likely to choose the larger delayed reward. Task type, age, and reward type did not significantly predict sex differences. Heterogeneity across studies was low and non-significant and no evidence of publication bias was found
Dutra	2016	SR MA	9 P	ADHD n = 217 Controls n = 244	Children Adolescents Adults	Mod	Children and adults with ADHD displayed short intracortical inhibition (a marker of motor inhibition). No differences in resting motor threshold and silent period were found. Significant statistical heterogeneity was detected between trials. Clinical and methodological heterogeneity may also have been present. Risk of publication bias not assessed
Ellison-Wright	2008	MA	7 P	ADHD n = 114 Controls n = 143	Children Adolescents Adults	Low	Individuals with ADHD displayed significant regional grey matter reduction the R putamen/globus pallidus region. Clinical heterogeneity and publication bias may be present; therefore, the possibility of grey matter decreases in the bilateral putamen/globus pallidum, caudate and frontal lobes cannot be ruled out. Risk of publication bias not assessed
Frodl	2012	SR MA	11 P	ADHD n = 320 Controls n = 288	Children Adolescents Adults	Mod	Children with ADHD displayed significantly reduced grey matter volumes in the R globus pallidus, R putamen and decreased bilateral caudate volumes. Adults with ADHD displayed significantly reduced anterior cingulate cortex volumes. Meta-regression indicated that studies in untreated children may also show changes in the anterior cingulate cortex and the amygdala region. Heterogeneity was not seen on the putamen and anterior cingulate cortex but was present in the globus pallidus and in the frontal, temporal, and parietal lobes that did not show significant differences. Risk of publication bias not assessed
Fusar-Poli	2012	MA	9 P	ADHD n = 169 Controls n = 173	Adults	Mod	Although striatal dopamine transporter density was 14% higher on average in the ADHD group, heterogeneity across studies was large and statistically significant. Pooled meta-analysis indicated consistent statistical evidence for greater dopamine transporter density in the ADHD group in the whole striatum (small effect size). Meta-regression analyses showed that the percentage of subjects without exposure to psychostimulants was negatively correlated with dopamine transporter density; density was higher in patients with previous medication exposure and lower in medication-naive patients. There was no moderating effect for age, comorbidity, gender, year of publication, or imaging technique. No evidence of publication bias found. Sensitivity analysis confirmed robustness of the results
Gao	2019	SR MA	21 P	ADHD n = 700 Control n = 580	Children Adolescents Adults	Mod	ADHD is characterised by hyperconnectivity between the frontoparietal network and regions of the default node network and affective network, and hypoconnectivity between the frontoparietal network and regions of the ventral attention network and somatosensory network. No significant between-group heterogeneity was found in the results for the default mode network and affective network. For the frontoparietal network results, significant between-group heterogeneity was detected in the superior frontal gyrus and the putamen. No evidence of publication bias found
Geburek	2013	MA	7 P	ADHD n = 166 Controls n = 161	Adolescents Adults	Low	Error negativity was significantly attenuated in adolescents and adults with ADHD when participating in event-related potential and behaviour performance tasks (medium effect size). Task type, age and proportion of males did not affect results and low heterogeneity was noted. Risk of publication bias not assessed. Error positivity was attenuated only during GoNogo tasks, however significant heterogeneity was detected. Adolescents and adults with ADHD responded slower during flanker tasks and made more errors during GoNogo and flanker tasks
Geiss	2023	SR	15 P	ADHD n = 424 Controls n = 422	Adults	Mod	Individuals with ADHD displayed atypical cardiovascular autonomic modulation, predominantly in the form of reduced sympathetic modulation, during tasks that require attention and/or response regulation or induce emotional stress. Clinical and methodological heterogeneity may influence results. Risk of publication bias not assessed
Groen	2013	SR	25 N	ADHD n = 257 Controls n = 253	Children Adolescents Adults	Low	Children and adults with ADHD often displayed increased risky performance during gambling tasks (the effect size ranged between small and large depending on the task). The risk is stronger for children/adolescents with ADHD than for adults with ADHD. Results were highly heterogenous. Comorbid ODD/CD appears to increase the risk. Risk of publication bias not assessed
Hart	2013	SR MA	34 P	ADHD n = 458 Controls n = 498	Children Adolescents Adults	Low	Children and adults with ADHD displayed significantly reduced activation for (1) inhibition in the R inferior frontal cortex, supplementary motor area, and anteri- or cingulate cortex, as well as striato-thalamic areas, and (2) attention (interference control) in the R dorso- lateral prefrontal cortex, posterior basal ganglia, and thalamic and parietal regions. Inhibition meta-analysis showed the supplementary motor area and basal ganglia were under activated solely in children with ADHD, while the inferior frontal cortex and thalamus were under activated solely in adults with ADHD. Risk of publication bias not assessed
Hart	2012	MA	11 P	ADHD n = 150 Controls n = 145	Adolescents Adults	Mod	Individuals with ADHD displayed significantly reduced activation in typical areas related to timing (i.e., L inferior prefrontal cortex/insula, cerebellum, and L inferior parietal lobe), and in areas that presumably reflect problems with deactivation of the default mode network. Comorbidity did not influence findings. Risk of publication bias not assessed
Hervey	2004	SR MA	33 N	Unable to determine	Adults	Low	Adults with ADHD displayed atypical neuropsychological functioning, most notably in the domains of attention, distractibility, response inhibition, and memory, consisting of working memory, short-term memory and verbal long-term memory (moderate effect size). A small effect size was also found for processing speed and motor speed. Clinical heterogeneity present. Risk of publication bias not assessed
Hokken	2023	ScR	35 N	ADHD n = 788 Controls n = 865	Children	Low	Overall, the literature was found to be inconclusive on accuracy performance during visual search tasks in children with ADHD. However, children with ADHD seem to be more at risk for visual search performance accuracy impairments compared to controls. Risk of publication bias not assessed
Hoogman	2019	C/S ENGIMA	-	ADHD n = 2246 Controls n – 1934	Children Adolescents Adults	High	Only children with ADHD displayed slightly reduced but statistically significant total cerebral cortex surface area, most notably in the superior frontal gyrus, lateral orbitofrontal cortex, posterior cingulate cortex, rostral anterior cingulate cortex and middle temporal gyrus. The effects were most prominent in the youngest children (4–9 years). Children with ADHD also displayed slightly reduced but statistically significant cortical thickness in 2 regions (fusiform gurus and temporal pole). These differences were most prominent in the group aged 10 and 11 years
Hoogman	2017	MA of C/S data	-	ADHD n = 1713 Controls n = 1529	Children Adolescents Adults	High	Children with ADHD displayed small but significant reductions in intracranial volume as well as significantly smaller nucleus accumbens, amygdala (largest effect size), caudate nucleus, hippocampus, and putamen volumes. These differences were not present in adults with ADHD. Explorative lifespan modelling suggested a delay of maturation and a delay of degeneration. Psychostimulant medication usage or the presence of comorbid psychiatric disorders did not influence results, nor did symptom scores correlate with brain volume
Hou	2023	SR	16 P	ADHD n = 321 Controls n = 334	Children Adolescents Adults	Mod	Children and adults with ADHD displayed smaller, weaker or inactive activation in the frontal cortex during working memory and inhibition control. No publication bias present, however, the following was detected: information bias (two included studies) and confounding bias (two included studies)
Huang-Pollock	2012	SR MA	47 N	ADHD n = 1181 Controls n = 1518	Children Adolescents	Mod	School aged children with ADHD displayed difficulty with (1) overall performance on tasks requiring sustained attention (large effect size) and (2) maintaining attention over time (small to medium effect size). They committed more omission and commission errors and displayed slower/more variable reaction times. Measurement unreliability accounted for a significant proportion of reported heterogeneity. Publication bias corrected during meta-analysis
Huizenga	2009	MA	41 N	ADHD n = 1540 Controls n = 1433	Children Adolescents	Mod	Inhibitory dysfunction in children and adolescents with ADHD, assessed by the stop-signal reaction time, is moderated by task complexity (stop signal reaction time difference significantly increased with increased with task complexity), and is most pronounced for spatially noncompatible responses. Participant age, medication status, percentage of female participants and number of learning trials did not affect findings. Risk of publication bias not assessed
Hulsbosch	2021	SR	19 N	ADHD n = 539 ADHD/ODD/CD n = 17 Controls n = 546	Children Adolescents	High	Children with ADHD did not display clear problems with basic instrumental learning under laboratory conditions, but may display problems when task complexity increases. Children displayed problems with conditional discrimination learning, a complex form of learning, particularly when there is a delay between the discriminative cue and the opportunity to respond. It is possible that they also display difficulties with reversal learning. No differences were found in regard to the effect of reinforcement on instrumental learning. Significant heterogeneity and some publication bias identified
Hutchinson	2008	MA	13 P	ADHD n = 284 Controls n = 311	Adolescents	Mod	Female children and adolescents with ADHD displayed significantly smaller splenium size (moderate effect size). Male children and adolescents with ADHD displayed significantly smaller rostral body size. Participant age did not affect findings. Cannot determine effect of comorbidity. Publication bias unlikely
Jackson	2016	SR MA	49 N	Participants n = 3913	Children Adolescents Adults	Mod	Delay discounting is significantly and robustly elevated among children and adults with ADHD (medium effect size). No significant differences based on sample age, reward outcome, or comorbid status was detected. Minimal heterogeneity or evidence of publication bias
Kaiser	2020	SR MA	53 P	ADHD n = 1576 Control n = 1794	Children Adolescents Adults	Mod	Children and adults with ADHD displayed on average atypical event rate potential during inhibitory control, attention, working memory, and performance monitoring. On average children and adults with ADHD displayed smaller Cue-P300-amplitudes, longer Go-P300-latencies, smaller NoGo-P300-amplitudes, longer NoGo-P300-latencies, smaller CNV-amplitudes, and smaller Pe-amplitudes. Children with ADHD: larger mean effect sizes for the NoGo-P300-amplitude, Pe-amplitude, Go-P100-latency, Go-P300- latency and NoGo-N200-latency. Adults: largest mean effect sizes for the Cue-P300-amplitude component. Results are characterised by substantial heterogeneity and moderate effect sizes
Kamradt	2018	SR MA	12 P	Participants n = 1041	Children Adolescents Adults	Mod	No effect was found between cortisol reactivity and ADHD, although significant heterogeneity may moderate this finding. No evidence of publication bias
Karalunas	2014	SR MA	18 N	ADHD n = 426 ASD n = 455 Control n = 1065	Children Adolescents	Mod	Children and adolescents with ADHD displayed significantly slower drift rates (moderate to large effect size), significantly faster nondecision times (small effect size). Findings underlie the reaction time variability differences displayed by individuals with ADHD. No evidence of heterogeneity or publication bias
Karalunas	2013	MA	7 P	ADHD n = 640 Controls n = 626	Children Adolescents	Low	Children with ADHD demonstrated more low-frequency reaction variability (small effect size) as well as equivalent excess reaction variability in a faster frequency comparison band (small effect size). There was a trivial and nonsignificant difference between effect sizes in the low and high frequency bands. New data replicated these results. Between study heterogeneity present. Publication bias corrected during meta-analysis
Kasper	2012	SR MA	45 N	ADHD n = 1989 Controls n = 1874	Children Adolescents	Mod	Children with ADHD exhibit heterogenous, statistically significant phonological and visuospatial working memory task deficits (large effect size). Several moderative variables (i.e., percentage of female participants, number of experimental trials, recall vs. recognition tasks, and demands on the central executive, explained the significant effect size variability among phonological and visuo-spatial studies. Mild evidence of publication bias within the sample of studies present
Koenig	2017	SR MA	8 P	ADHD n = 317 Controls n = 270	Children Adolescents Adults	Mod	Children and adults with ADHD did not display altered short-term measures of resting-state vagal tone. No significant heterogeneity across effect sizes reported. Risk of publication bias not assessed
Kofler	2013	SR MA	319 N	ADHD n = 9780 Controls n = 12,024	Children Adolescents Adults	High	Children with ADHD demonstrated robust but heterogenous impairments in reaction time variability relative to controls (large effect size). This increased variability was attenuated by psychostimulant treatment but unaffected by non-stimulant medical and psychosocial interventions. Adults with ADHD continue to demonstrate significantly increased reaction time variability (moderate effect size). Processing speed was unaffected when reaction time variability is accounted for. Publication bias corrected during meta-analysis
Kowalczyk	2022	SR	34 P	ADHD n = 981 ADHD remit n = 38 Non-ADHD sibling n = 134 Controls n = 774	Children Adolescents Adults	Mod	Across cognitive domains, children and adults with ADHD consistently displayed atypical functional connectivity in the cingulo-opercular, sensorimotor, visual, subcortical, and executive control networks, and similar sensorimotor and subcortical (primarily striatal) networks. Large heterogeneity in study methodologies prevented a meta-analysis. Low risk of publication bias
Lanier	2021	SR	11 N	ADHD n = 291 Controls n = 197	Children Adults	Low	ADHD is commonly associated with impairing spontaneous mind wandering. Risk of publication bias not assessed
Lansbergen	2007	SR MA	18 N	ADHD n = 757 Controls n = 605	Children Adolescents Adults	Low	Children and adults with ADHD consistently displayed significantly compromised interference control. Small effect size deemed due to between study methodological differences and an additional random component. Risk of publication bias not assessed
Lei	2015	MA	23 P	ADHD n = 324 Controls n = 350	Children Adolescents Adults	Mod	Individuals with ADHD displayed significantly decreased activation during response inhibition in the supplementary motor area, insula, caudate, and precentral gyrus, as well as increased activation in the postcentral gyrus, inferior frontal gyrus, and precuneus. The activation decreases in the R caudate were greater in child ADHD patients than adult ADHD patients. Risk of publication bias not assessed
LeRoy	2019	SR	16 N	ADHD n = 718 Controls n = 337	Adults	Low	Adults with ADHD-C and ADHD-I performed worse on measures of executive functioning (tests measured cognitive inhibition, selective attention, cognitive flexibility, processing speed, planning), attention (tests measured orientating attention, response time, vigilance), working memory, and memory. Direct comparisons of the two subtypes showed that those with ADHD-C performed worse in the memory domain. There were few to no differences in other domains. Risk of publication bias not assessed
Lijffijt	2005	MA	29 N	ADHD n = 977 Controls n = 1078	Children Adolescents Adults	Low	Children with ADHD but not adults with ADHD display significantly longer basic reaction time (moderate effect size). Adults with ADHD display a slowing of stop sign reaction time relative to mean reaction time (moderative effect size). Risk of publication bias not assessed
Liu	2023	MA	38 P	ADHD n = 1,126 Controls n = 1277	Children Adolescents Adults	Mod	Children with ADHD consistently exhibited hyper-connectivity between different parts of the cortex and L middle frontal gyrus, and hypo-connectivity between different parts of the cortex and L putamen/pallidus/ amygdala. Adults with ADHD show hyper-connectivity between the cortex and R precuneus/sub-gyral/cingulate gyrus. Risk of publication bias not assessed
Loyer-Carbonneau	2021	MA	54 N	ADHD n = 10,212	Children Adolescents	High	Boys and girls with ADHD presented with significantly more executive (i.e., working memory, planning, cognitive flexibility, motor response inhibition, and interference control) and attentional (i.e., sustained, selective and divided attention) deficits. Boys with ADHD tend to display more hyperactivity as well as more difficulties with response inhibition and cognitive flexibility in comparison to girls with ADHD. Significant heterogeneity between effect sizes noted. Effect sizes did not seem to be affected by a publication bias
Lukito	2020	SR MA	80 P	ADHD n = 2534 Controls n = 2299 ASD n = 60	Children Adolescents Adults	Mod	ADHD is associated with reduced grey matter volume and under activation in a number of brain regions during cognitive control and motor response inhibition. Findings somewhat vary between children and adults with ADHD. No evidence of publication bias
Ma	2016	MA	10 P N	ADHD n = 210 Controls n = 274	Children Adolescents	Low	Reinforcement can normalise inhibitory control in children and adolescents with ADHD (large effect size). Inhibitory control may also improve to a larger extent in adolescents with ADHD as a function of reinforcement. Potential factors contributing to the reinforcement effects include the reinforcement schedules and the type of reinforcement. Heterogeneity deemed low. Risk of publication bias not assessed
Marx	2021	SR MA	37 N	Participants n = 3763 (53% with ADHD)	Children Adolescents Adults	Mod	Studies examining single choice paradigm: Individuals with ADHD tended to choose small immediate rewards over large delayed rewards more frequently than controls (small to medium effect size). Significant interstudy heterogeneity and possible publication bias noted. Studies examining temporal discounting: offering real rewards to individuals with ADHD nearly halved choice impulsivity in participants with ADHD. No evidence of heterogeneity and publication bias
Mauri	2018	StR	11 P	ADHD n = 196 Controls n = 195	Children Adolescents	Very Low	Children and adolescents with ADHD tended to displayed peculiar cortical activation both during neurological and emotional tasks, and lower (1) prefrontal cortex activation during tasks assessing attention, cognitive inhibition and flexibility, working memory and memory span, and (2) temporal cortex re-activity to facial emotional stimuli. Consistent interpretation of findings is limited due to substantial methodological heterogeneity. Risk of publication bias not assessed
McCarthy	2014	SR MA	20 P	ADHD n = 334 Controls n = 372	Adolescents Adults	Mod	Across all tasks, adolescents and adults with ADHD displayed significantly less frontal lobe activity. N-back task: less activity in the bilateral superior frontal gyri and L medial frontal gyrus. Go/no-go tasks: less mean activation in the L medial frontal gyrus and R caudate. Stop tasks: children displayed less activity the bilateral inferior frontal gyri, R superior frontal gyrus and R middle frontal gyri. Risk of publication bias not assessed
Metin	2012	MA	30 N	ADHD n = 2091 Controls n = 3836	Children Adolescents Adults	Low	Children and adults with ADHD displayed significant and disproportionate slowing of reaction time on trials with slow event rates. For commission errors, the effect sizes were larger on trials with fast event rates. No event-related effects were seen for reaction time variability. There were also general effects of ADHD on performance for all variables that persisted after effects of event-rate were taken into account. Significant heterogeneity account for by using a random effects model. Risk of publication bias not assessed
Mette	2023	StR	7 N	ADHD n = 346 Controls n = 286	Adults	Low	The results of the present review indicate that the number of studies on time perception in adult ADHD is very scarce. Moreover, the main investigated domains of time perception in the past decade were time estimation, time reproduction and time management. Whereas some of the found studies were able to demonstrate a distinct deficit in time estimation, time reproduction and time management other studies were unable to demonstrate a clear association between ADHD and time estimation and time reproduction deficit. Significant clinical and methodological heterogeneity reported. Risk of publication bias not assessed
Michelini	2022	SR MA	28 P	MA ADHD n = 645 MA Controls n = 521	Children Adolescents Adults	Mod	Children and adults with ADHD displayed broad brain-oscillatory alterations with small (theta) and small-to-moderate (alpha and beta) effect sizes. These group differences were partly consistent when repeating analyses by age group (< 18 and 18 + years) and task type (cognitive control, working memory, and simple attention tasks). Risk of publication bias not assessed
Mills	2018	LS	P	ADHD n = 256 Controls n = 176	Adolescents	High	Children with ADHD displayed reduced negative connectivity between task positive (i.e., fronto-parietal, cingulo-opercular, and dorsal attention networks), and task negative networks (i.e., default mode network). Connectivity continues to become more negative throughout development. Children with ADHD displayed poorer signal detection on the continuous performance task (reduced attentional vigilance), more so on easy than difficult tasks
Mowinckel	2015	SR MA	59 N	Unable to determine	Adults	Mod	Adults with ADHD displayed atypical decision-making. Reward based decision making and continuous performance task: small to medium effect sizes. Drift diffusion model analysis: large effect size. No evidence of publication bias
Mullane	2009	SR	12 N	ADHD n = 272 Controls n = 280	Children	Low	Children with ADHD displayed significantly poorer interference control as indexed by reaction time (results were heterogenous), accuracy (large effect size), and inverse efficiency congruency (large effect size). Risk of publication bias not assessed
Mullane	2008	StR	7 N	ADHD n = 180 Controls n = 193	Children Adolescents	Low	Children and adolescents with ADHD demonstrated less efficient serial search, both at the lowest and highest levels of task complexity, with difficultly increasing with demand (i.e., increased search complexity and large item numbers). Results were variable for effortful serial search. No differences in automatic search were found. These findings indicate children with ADHD show impairments in aspects of effortful visual selective attention, as measured by visual search. Risk of publication bias not assessed
Nakao	2011	SR MA	14 P	ADHD n = 378 Controls n = 344	Children Adolescents Adults	Low	Children and adults with ADHD displayed significantly smaller grey matter volume. This difference was more pronounced when the adult study was excluded. No significant heterogeneity or publication bias noted. Children with ADHD may display significantly and robustly smaller grey matter volumes in the R lentiform nucleus, extending to the caudate, and larger grey matter volumes in the L posterior cingulate cortex. Findings were heterogenous
Nejati	2020	MA	25 N	ADHD n = 1660 Controls n = 1234	Children	High	Children with ADHD displayed significant difficulties in time perception. The problem is most pronounced in tasks of longer duration but also obvious during tasks assessing different types of modalities (i.e., visual and auditory), and time estimation, reproduction and anticipation. Heterogeneity in the published studies was large and statistically significant. No evidence of publication bias
Onandia-Hinchado	2021	SR	93 N	ADHD n = 5574 Controls n = 4880 Other n = 1323	Adults	Low	Adults with ADHD displayed atypical attention regulation (alertness, selective and sustained), processing speed, executive function (mainly working memory and inhibition with emphasis on reward delay and interference control), verbal memory, reading skills, social cognition and arithmetic abilities. Risk of publication bias not assessed
Oosterlaan	1998	MA	8 N	ADHD n = 180 CD/ODD n = 68 ADHD/CD/ODD n = 65 Controls n = 159 Other n = 74	Children	Low	Children with ADHD displayed poor response inhibition related to a slow inhibitory process during tasks measuring Go Response Mean Reaction Time (medium effect size), Inhibition Function Slope (large effect size), and Stop Signal Reaction Time (medium effect size). The findings for children with conduct disorder were similar but less consistent. Children with ADHD and conduct disorder display greater impairments than children with only ADHD. Risk of publication bias not assessed
Orban	2022	MA	20 N	ADHD n = 641 Controls n = 557	Children Adolescents Adults	High	Across all studies examining constructs of memory control, individuals with ADHD demonstrated worse control over memory and differences, along with differences in verbal memory interference control during the following measurement tasks: continuous moderators of practice interference (children only, moderate effect size, heterogeneity corrected during meta-analysis) and retroactive interference (children and adults, non-significant heterogeneity). Homogeneity was significant. No evidence of publication bias
Paloyelis	2007	SR	20 P	Unable to determine	Children Adolescents Adults	Very Low	Individuals with ADHD display both lower and higher brain activity in different regions of the brain dependent on task. In analyses that examined inhibition errors, as well as in tasks that tapped attention processes, motor function and working memory, the ADHD group almost exclusively showed lower activity. In the attentional tasks, this was mostly over temporal and parietal areas; in motor function tasks, mostly over frontal areas. Risk of publication bias not assessed
Patros	2019	SR MA	41 N	ADHD n = 2051 Controls n = 2766	Children	High	Children with ADHD experienced significant atypical planning (moderate effect size) and responded more quickly on planning tasks (small to moderate effect size). Participant age, percentage of females, solution presentation (e.g., pictorial vs. physical display), and task complexity (beads vs. disks) were identified as statistically significant moderating variables across planning metrics. No evidence of publication bias noted in the results pertaining to planning accuracy, composite planning performance, rule violations, problem-solving time and initiation time when baseline motor-response time controlled. The potential for publication bias was noted in regard to the initiation time and solution time results
Patros	2016	SR MA	26 N	ADHD n = 2360 Controls n = 1960	Children Adolescents	High	Children and adolescents with ADHD exhibited increased impulsive decision-making (moderate effect size). When choice-impulsivity tasks were examined independently, children with ADHD performed significantly more impulsively on both delay of gratification and delay discounting tasks. The use of single-informant diagnostic procedures relative to multiple informants yielded larger between-group effects, and a similar pattern was observed across samples that excluded females relative to samples that included females. No evidence of publication bias noted in the results pertaining to choice impulsivity and delay of gratification. The potential for publication bias was noted in regard to the delay discounting results
Pauli-Pott	2015	MA	19 N	ADHD n = 1395 Controls n = 1195	Children Adolescents	Mod	The executive inhibitory control and delay aversion/discounting challenges children with ADHD experience during complex cool inhibition tasks (which specifically involve the executive attention system) increased with age between 3 and 6 years (moderate effect size), but appear to become smaller, although still significant (small effect size) when studies include adolescents aged between 13 and 16 years. There was no significant difference between studies that used versions of the delay/temporal discounting task vs. other tasks. Results were not influenced by proportion of boys and co-morbidity. Risk of publication bias not assessed
Pievsky	2018	SR of MA	34 MA N	Unable to determine	Children Adolescents Adults	Mod	ADHD is associated with heterogenous difficulties across a variety of neurocognitive including set shifting, working memory reaction time variability response inhibition, vigilance, intelligence/achievement and planning/organisation (all moderate effect size except for set shifting which had a small effect size). Risk of publication bias corrected during meta-analysis. Age moderated the relationship between ADHD diagnosis and neurocognitive functioning, with greater between-groups differences among children and adults than among adolescents. Studies that received drug funding reported larger effect sizes than those without
Plichta	2014	MA	10 P	ADHD n = 231 Controls n = 185	Children Adolescents Adults	Very low	Individuals with ADHD displayed ventral-striatal hypo-responsiveness during task measuring reward anticipation (moderate effect size). The included studies were homogenous. Risk of publication bias not accessed
Posner	2014	SR	24 P	ADHD n = 583 Controls n = 927	Children Adolescents Adults	Very Low	ADHD is associated with heterogenous anticorrelations (1) between the cognitive control network and the default mode network, (2) within the default node network and the cortioco-striato-thalamo-cortical loops (cognitive and limbic). Risk of publication bias not assessed
Proal	2011	LS	-	ADHD n = 207 Mean age at entry 8 years Controls n = 178	Children Adolescents	High	Adults diagnosed with childhood ADHD displayed significantly (1) reduced cortical thickness in the dorsal attentional network and limbic areas (emotional regulation and motivation), and (2) decreased grey matter in the R caudate, R thalamus and bilateral cerebellar hemispheres (R hemisphere clusters were located in the inferior parietal lobe, temporal pole, and insula; L hemisphere clusters were located in superior frontal gyrus/frontal pole, precentral gyrus, insula, temporal pole, and cuneus). They also displayed cortical thinning in the bilateral parietal lobes, occipital lobe, temporal poles, insula, precentral gyri, frontal poles, and R precuneus
Ramos	2020	SR MA	49 N	ADHD n = 4956 Controls n = 3249	Children Adolescents	Mod	Children and adolescents with ADHD exhibited robust poorer working memory performance during digital scan backwards performance (medium effect size). Effect size reduced with increasing age and explained a moderate level of heterogeneity, with younger children presenting with the greatest difficulties. A small level of publication bias noted
Rash	2012	SR	6 P	ADHD n = 155 Controls n = 89	Children Adolescents	Low	Findings were not unanimous but suggest children with unmedicated ADHD experienced lower levels of cardiac vagal tone during tasks that involved self-regulation and emotion regulation. Medication acted to correct the autonomic imbalance experienced by children with ADHD but did not bring this imbalance into normal levels. Risk of publication bias not assessed
Robe	2019	SR MA	13 P	ADHD n = 869 Controls n = 909 Children, adolescents	Children Adolescents Adults	High	Individuals with ADHD displayed heterogenous reduced vagally-mediated heart rate variability post task demand (small affect size). Effect size was greatest in the presence of co-morbidity. Task type and respiration rate also significantly moderated findings. Significant risk of publication bias noted
Roberts	2021	MA	116 N	Unable to determine	Children Adolescents Adults	Mod	Children and adults with ADHD exhibit more risk-taking compared to children and adults without the disorder (moderate effect size). Larger effect sizes were associated with studies involving virtual reality simulations and those in which risky options were disadvantageous, compared to studies in which risky options were more advantageous or similar to safe alternatives. No evidence of publication bias was noted for results related to self-reported measures and virtual reality tasks. For studies encompassing behaviour tasks publication bias may be present
Samea	2019	SR MA	96 P	n = 1914	Children Adolescents	Mod	Pooled structural and functional, sub-analyses restricted to modality, and in-/decreased contrast did not yield any significant finding. Sub-analysis of task t-fMRI experiments using neutral stimuli resulted in convergence of aberrant ADHD-related finding in the L pallidum/putamen. Decreased activity in the L inferior frontal gyrus was also found in male subjects only. Lack of regional convergence in other areas in children/adolescents with ADHD may be due to heterogeneous clinical populations, various experimental design, pre-processing, and statistical procedures in individual publications. Risk of publication bias not statistically assessed
Sanjeevan	2020	SR MA	7 N	ADHD n = 213 Controls n = 257	Children Adolescents Adults	High	Procedural sequence learning appears to be preserved in ADHD. Heterogeneity was significant across studies and could be partially attributed to the age of participants. No evidence of publication bias
Schoechlin	2005	SR MA	24 N	ADHD n = 867 Controls n = 806	Adolescents Adults	Mod	In all functional domains, adults with ADHD scored significantly lower in the domains of visual and verbal fluence, abstract problem-solving, sustained attention, focused attention, verbal memory (all moderate effect size), as well as verbal intelligence (reasoning), executive functions, visual/figural problem-solving, simple attention and figural (visual) memory (all small effect size). Heterogeneity was only significant for visual/verbal fluency and simple attention. Risk of publication bias deemed unlikely
Schwartz	2008	MA	25 N	ADHD n = 1535 Controls n = 1730	Children Adults Adolescents	Mod	Response inhibition measured using the Stroop interference effect (the tendency to experience difficulty naming a physical colour when it is used to spell the name of a different colour) was not larger in children or adults with ADHD regardless of age. Children and adults with ADHD however responded on average 1.14 times slower during both the colour and the colour-word condition. Risk of publication bias not assessed
Senkowski	2022	SR MA	26 N	ADHD n = 883 Controls n = 916	Adults	High	Adults with ADHD display reliable inhibitory control deficits, as expressed in prolonged stop signal task response times (moderate effect size). Results were not moderated by study quality, sample characteristics or clinical parameters. Adults with ADHD may also display greater stop signal task omission errors and reduced go accuracy (small effect size), indicative of altered sustained attention. Subtle publication bias may be present
Shaw	2013	LS	P	ADHD n = 92 Controls n = 184	Adolescents Adults	High	Adult ADHD status is associated with altered development and increased cortical thinning of components of the cortical networks and support attention, cognitive control, and the default mode network, Specifically, the R cingulate gyrus (extending to the R caudal regions and R isthmus, and extending to the R precuneus of the superior parietal lobe, R cuneus of the occipital lobe, and R fusiform gyrus), R dorso-lateral prefrontal cortex, L caudal anterior cingulate, L para-central lobule, L precuneus, and L postcentral gyrus
Shaw	2012	LS	P	ADHD n = 234 Controls n = 231	Children	High	Although childhood maturation of the cortical surface area progressed along the typical trajectory, surface area development was delayed in children with ADHD. When children first participated in the study (mean age 10.4 years), cortical surface reduction was most pronounced bilaterally in the prefrontal cortex (especially the medial wall and the lateral superior, middle, and polar frontal regions), the R lateral temporal cortex, and L medial temporal cortex, extending to posterior L medial wall (fusiform, lingual gyri, and cuneus). At a sub-lobular level, delay was most prominent in the anterolateral prefrontal gyri bilaterally, especially on the right. Additionally, delay was prominent bilaterally in the medial temporal gyri, in the R postcentral and middle temporal gyri, and L supramarginal gyrus. For the R prefrontal cortex, the median age by which children with ADHD achieved attained peek area in 50% of cortical vertices was 14.6 years, significantly later than controls at 12.7 years. A similar, but less pronounced, delay was found in the L hemispheric lobes. There were no such diagnostic differences in the developmental trajectories of cortical gyrification
Shaw	2007	LS	P	ADHD n = 223 Controls n = 223	Children	High	Although regional brain maturation progresses in a typical manner, children with ADHD displayed marked delay in attaining peak thickness throughout most of the cerebrum. The mean age at which children with ADHD attained peak thickness for 50% of the cortical points was 10.5 years, which is significantly later than the mean age of 7.5 years for controls. Differences were most prominent in the middle prefrontal cortex, where the ADHD group reached their peak thickness approximately 5 years after controls, and to a lesser extent in the superior prefrontal and medial prefrontal cortex. Delay was also found in the temporal cortex extending to the middle occipital gyri, most prominently in the posterior portions of the middle and superior temporal gyrus bilaterally
Shaw	2006	LS	P	ADHD n = 163 Controls n = 166	Children	High	Children with ADHD displayed global cortical thinning, most prominently in the medial and superior prefrontal and precentral regions, including the superior and medial frontal gyri and cingulate region bi-laterally, L precentral gyrus, and R anterior/mesial temporal cortex. Cortical thickness developmental trajectories did not differ significantly between participants with ADHD and control groups throughout except in the R parietal cortex, where trajectories converged by age 17
Skodzik	2017	SR MA	24 N	ADHD n = 827 Controls n = 771	Adolescents Adults	Mod	Adults with ADHD displayed significant verbal working memory deficits (medium effect size) but not visual working memory deficits during tests examining delayed free recall, memory acquisition and recognition memory. Results were heterogeneous. No evidence of publication bias. The long-term memory performance problems adults with ADHD experience are strongly influenced by deficits already present in the stage of memory acquisition and reflects a learning (memory acquisition) challenge induced at the stage of encoding
Snyder	2006	MA	9 P	n = 1498	Children Adolescents Adults	Mod	Children and adults with ADHD displayed a consistent increase in theta/beta ratio (medium effect size). Heterogeneity was attributed to (1) meta-analytic technique, (2) eyes open versus eyes closed, and (3) control of medication. Risk of publication bias not assessed
Sutcubasi	2020	SR MA	20 P	ADHD n = 944 Controls n = 1121	Children Adolescents Adults	Mod	Children and adults displayed within-default mode network hypo- and hyper-connectivity, and within-cognitive control system hyperconnectivity. Children and adolescents also displayed reduced connectivity between the default mode and cognitive control networks, and the affective/motivational and salience networks. Risk of publication bias not assessed
Szuromi	2011	SR MA	6 P	ADHD n = 154 Controls n = 140	Adolescents Adults	Low	Adults with ADHD displayed significantly reduced P3 amplitude in comparison to controls (medium effect size). The effect size is greater in females than males, and the reduction in P3 amplitude appears to increase with age. Risk of publication bias not assessed
Talbot	2018	SR	6 N	ADHD n = 162 Controls n = 176	Children Adolescents	Low	Children with ADHD performed significantly worse on time-based prospective memory tasks and event-based prospective memory tasks (both considered a type of long-term memory or involving long-term memory), irrespective of their ongoing task performance. Risk of publication bias not assessed
Valera	2007	MA	21 P	ADHD n = 565 Controls n = 583	Children Adolescents	Mod	Children with ADHD displayed heterogenous global volume reductions, the largest differences include cerebellar regions, the splenium of the corpus callosum, total and R cerebral volume, and R caudate. Risk of publication bias not statistically assessed
Valmiki	2021	SR	9 N	ADHD n = 493 Controls n = 330	Adolescents (male only)	Mod	Male adolescents with ADHD are more responsive to reinforcement related to monetary gain and loss and are more sensitive to larger rewards than smaller ones. ADHD-C and typically developed controls were found to have similar responsiveness, while ADHD-I appeared to be insensitive to stimulus. Risk of publication bias not assessed
Van Ewijk	2012	SR MA	15 P	ADHD n = 173 Controls n = 169	Children Adolescents Adults	Mod	Children and adults with ADHD displayed wide spread alterations in white matter integrity, most evidently in the R anterior corona radiata, R forceps minor, bilateral internal capsule, and L cerebellum. Other areas that were less reliably implicated include the inferior and superior longitudinal fasciculus, cingulum, corpus collosum, caudate, basal ganglia, uncinate fasciculus, forceps minor, frontal, temporal, parietal and occipital lobes. Risk of publication bias not assessed
Vidor	2022	SR MA	33 P	ADHD n = 874	Children	High	Children with ADHD displayed higher concentrations of a composite of glutamate and glutamine in the R medial frontal area. Glutamate may be implicated in pruning and neurodegenerative processes as an excitotoxin, while glutamine excess might signal a glutamate depletion that could hinder neurotrophic activity. Both neuro metabolites could be implicated in the differential cortical thinning observed in patients with ADHD across all ages. Risk of publication bias not assessed
Vieira de Melo	2018	SR	6 P	n = 4144	Children Adolescents Adults	Low	Childhood ADHD is associated with overall and region-specific cerebral volume reductions, and hypo- and hyper-activation. Adults with ADHD displayed region-specific frontal lobe hypoactivation and parietal lobe hyperactivation. It was impossible to determine if age, gender, and comorbidity accounted for heterogeneity. Risk of publication bias not assessed
Willcutt	2005	MA	83 N	ADHD n = 3734 Controls n = 2969	Children Adolescents Adults	Low	Children and adults with ADHD displayed significant weaknesses on all executive function measurements (medium effect size). The strongest and most consistent effects were obtained on measures of response inhibition, vigilance, working memory, and planning. Adjusting for heterogeneity did not influence effect size. Effects could not be attributed to variables such as intelligence, reading ability, and symptoms of other disorders. Risk of publication bias not assessed
Yap	2021	SR	31 P N	ADHD n = 667 Controls n = 698	Children Adolescents Adults	Mod	Individuals with ADHD displayed activation alterations in the frontal and parietal regions and poorer sustained attention and working memory, regardless of age and task paradigm. Functional heterogeneity could be attributed to different brain activation patterns, large intra-subject variability, or both. Risk of publication bias not assessed
Zhang	2023	MA	47 P	ADHD n = 1150 ASD n = 648 Controls n = 1615	Children Adolescents Adults	High	Children with ADHD do not display significant fractional anisotropy changes. Adolescents with ADHD displayed decrease fractional anisotropy in the corpus callosum (body and splenium), L optic radiations, R superior longitudinal fasciculus, L pons, inferior longitudinal fasciculus, and R corticospinal projections. Adults with ADHD displayed increased fractional anisotropy in the L superior longitudinal fasciculus, body of the corpus callosum and R precuneus as well as decreased fractional anisotrophy clusters in the genu, body and splenium of the corpus callosum
Zhang	2018	SR MA	10 P	ADHD n = 654 Control n = 529	Children Adolescents	High	Only males with ADHD displayed significantly higher peripheral brain-derived neurotrophic factor levels (a key molecule involved in differentiation of neuronal populations during development and plastic changes related to learning and memory). High heterogeneity was noted across sampled studies, which may be a function of sample size, participants sampled, variations in study design, or other factors. Risk of publication bias not assessed
Zhao	2022	MA	66 P	n = 4137	Children Adolescents Adults	High	Children and adults with ADHD displayed decreased fractional anisotropy. Decreased fractional anisotropy was identified in one cluster in the corpus callosum via anisotropic effect size-signed differential mapping. Results may be affected by potential publication bias. Decreased fractional anisotrophy was identified at the genu, splenium of the corpus callosum and parietal lobe via activation likelihood estimation. No evidence of publication bias. Findings indicate ADHD is associated with the presence of aberrant white matter microstructure
Zheng	2022	MA	27 N	ADHD n = 1620 Controls n = 1249	Children Adolescents	High	Children and adolescents with ADHD perceived time less accurately (medium effect size) and less precisely (medium effect size) and displayed a higher tendency to overestimate time than controls. Moderator analyses indicated that the discrepancy of time perception between groups was not affected by the type of timing tasks nor the modality of stimuli used in the tasks, but was influenced by participant age with time perception abilities improving in terms of both accuracy and precision, with increased age. Low risk of publication bias for all results reported

Note:* SR* Systematic review,* MA* Meta-analysis,* StR *Structured review,* IR* Integrative review,* ScR *Scoping review,* LS *Longitudinal study,* C/S* Cross sectional;* P* Psychophysiological and brain-imaging study,* N* Neuropsychological study;* R* Right,* L *= Left;* TBSS* = Tract-based spatial statistics,* WBVBA* = Whole-brain voxel-based morphometry.

Data was then compared, categorised and grouped together in tables ready for analysis, as per the integrative review data analysis process outlined by Whittemore & Knafl [17] (see Supplementary material). Three overarching themes were identified: Delayed cortical maturation and atypical neuroanatomy, Atypical brain function, and Atypical cognitive abilities. For the atypical cognitive abilities theme, nine sub-themes were also identified. Data commonalities and differences where identified using an inductive analytic approach, the number of studies and qualitative scores contributing to a theme considered, and generalised, conceptual conclusions drawn. A narrative synthesis of the findings is presented below.

## Results

**Fig. 1:**
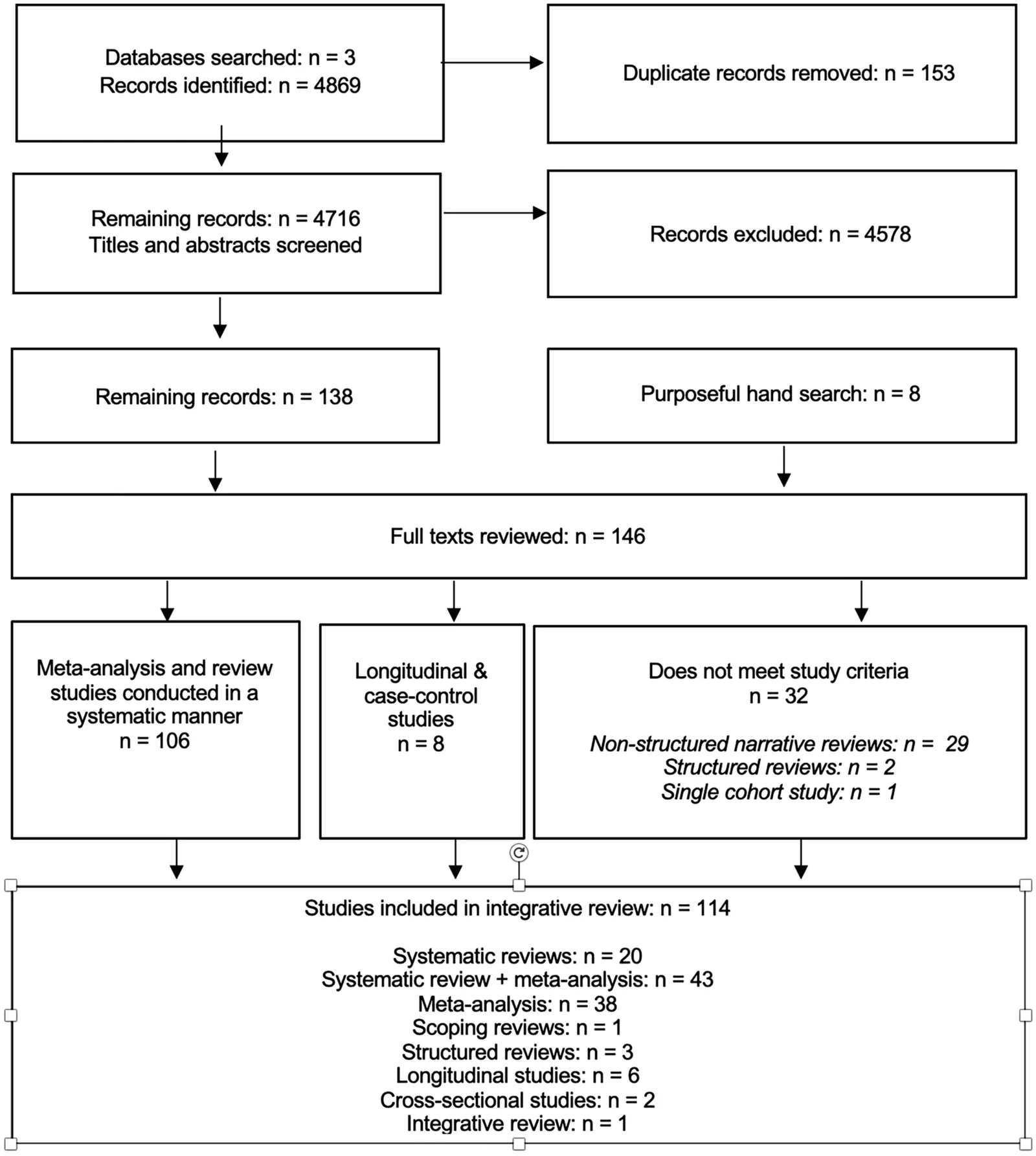
Adapted PRISMA review process flow

The search yielded 4869 papers, of which 153 were duplicates and removed. Following title and abstract screening, 4578 papers were excluded as they did not meet the study inclusion criteria. The six longitudinal case–control and two large cross-sectional neuroimaging studies identified by LEB were then added to the papers requiring full text screening bringing the total to 146; 114 papers were found to met the study inclusion critera (see Fig. 1). Supplementary information, Appendix C contains a list of excluded studies.

Table 1 contains a list of the review papers included in this integrative review, along with a summary of their findings and quality scores. Where available, reported magnitude of effect sizes are also reported in this table. Given the vast heterogeneity (i.e., speciality, inclusion/exclusion criteria, measures and constructs assessed; participant comorbidity), we do not report overall effect sizes in the narrative synthesis below. Instead, we use terms such as (1) tend to, and (2) possibly, potentially, may, etc., to reflect the number of review papers and their quality scores that were converged to contribute to an identified theme or the components of a theme, and the reported sample size, magnitude of effect sizes, heterogeneity, risk of bias, etc. Doings so aligns with integrative review methodology and our goal of creating a framework/checklist that is reflective of ADHD-related individuality and the limitations of study findings, which can be used to consider the factors that may be contributing to the challenges a person with ADHD may be experiencing. A composite summary of the allocated quality scores can be found in Supplementary information, Appendix D. Thirty-two review papers were ranked as high quality, 46 as moderate, 31 as low and five as very low. The main criteria that affected quality scores was lack of quality assessment (n = 89). Risk of publication bias was only assessed in 42 papers. As the impact of comorbidity on review study findings can only be determined via meta-analysis, systematic, scoping, and structured reviews were automatically marked ‘No’ for this criteria. Although a wide range of comorbid conditions could impact findings, if participants with at least one comorbid condition were excluded from studies employing meta-analysis, the need to statistically determine the impact of comorbidity was deemed ‘Not applicable’.

Sixty-two reviews included psychophysiological and brain-imaging studies, 48 neuropsychological studies, and four, a combination of both. The most common brain imaging methods employed were fMRI, closely followed by magnetic resonance imaging (MRI) (See Supplementary information, Appendix E). A wide variety of neuropsychology tests were employed to examine various aspects of cognition, such as working memory, response inhibition, attention regulation, cognitive flexibility, cognitive fluency, reaction time, etc., which made it difficult to consolidate results.

### Overview of Findings

Overall, findings converge to indicate ADHD is associated with (1) a significant delay in cortical maturation along with differences in neuroanatomy that do not appear to fully resolve in adulthood, (2) atypical brain function, and (3) atypical cognitive processes that link to the atypical neuroanatomy and brain function findings. The cognitive processes implicated include working memory, inhibitory control (response inhibition and attentional inference control), cognitive flexibility, alerting attention, reward processing, and long-term memory, along with reaction time, time perception and estimation, planning, and complex decision-making/problem-solving. The evidence for each of these findings is provided below.

### Delayed Cortical Maturation and Atypical Neuroanatomy

The findings reported in seven papers included in Table 1 (See Supplementary information, Appendix F), converge to indicate ADHD is possibly associated with a delay in childhood brain maturation, potentially by 2–3 years. Overall cerebral cortex [24] and surface area [21]; intercranial, striatal [20] and grey matter volume [25, 27]; along with cortical thickness has been found to fall outside the range expected according to age in children with ADHD [28, 29]. Shaw et al. [29] reported that children with ADHD achieve peak thickness in 50% of right frontal cortical vertices at a median age of 14.6 years, almost two years later than their typically developing peers. Similarly, Shaw et al. [28] reported children with ADHD attain peak cortical thickness in the primary sensory areas approximately three years after healthy peers. This delay in cortical maturation was most prominent in the lateral pre-frontal cortex which is associated with a range of cognitive functions including inhibitory control (response inhibition and attentional interference control), working memory, and reward processing [28]. Maturation of the bilateral middle and superior temporal cortices also appeared to be delayed by approximately 4 years [28].

In addition, findings of 17 studies (see Supplementary information, Appendix G) indicate children with ADHD display atypical neuroanatomy. This is possibly in the form of smaller amygdala surface area, particularly in the left hemisphere, which is involved in emotion processing [30]; and reduced grey matter volume in the (1) ventromedial frontal cortex (subcallosal gyrus), which has been implicated in reward-based decision-making [31], (2) right caudate, which is involved in inhibitory control and procedural and associative learning, (3) right thalamus, (4) bilateral cerebellar hemispheres [32], and (5) splenium [33]. Children with ADHD may also display higher concentrations of a composite of glutamate in the right medial frontal area, which has been implicated in neural pruning [34]. Furthermore, cortical thinning appears to be increased in children and adults with ADHD [35, 36] in the brain regions governing attention and emotional regulation. This includes the medial and superior prefrontal and precentral regions (superior and medial frontal gyri and cingulate region bilaterally, left precentral gyrus), right anterior/mesial temporal cortex [35], bilateral parietal lobes, and insula [32]. While males with ADHD possibly display significantly higher peripheral brain-derived neurotrophic factor levels, a key molecule involved in differentiation of neuronal populations during development and plastic changes related to learning and memory, findings were highly heterogeneous [37].

In line with nine studies (Supplementary information, Appendix H), although brain maturation in children with ADHD appears to progress in a similar manner to children without ADHD, and differences in cortical thickness and surface area appear to resolve by adulthood [38], many of the above ADHD-related neuroanatomical differences may not completely resolve in adulthood. Evidence of this includes, adults with ADHD possibly displaying reduced grey matter volumes in the ventromedial frontal cortex (subcallosal gyrus), which is implicated in risk, fear and reward-based decision-making [31], and the anterior cingulate cortex [25], which is implicated in reward processing, motivation, conflict and error monitoring and decision-making [39, 40]. They may also display differences in components of the cortical networks that support attention and cognitive control, [36], and the default mode network, which mediates introspective and self-referential cognition and mind wandering [40]. This includes, but is not limited to, the right cingulate gyrus (extending to the right caudal regions and right isthmus, and extending to the right precuneus of the superior parietal lobe, right cuneus of the occipital lobe, and right fusiform gyrus); right dorso-lateral prefrontal cortex, left caudal anterior cingulate, left para-central lobule, left precuneus, and left postcentral gyrus [36].

### Atypical Brain Function

Findings from 45 papers included in Table 1 (see Supplementary information, Appendix I) converge to indicate that ADHD is associated with atypical white matter microstructural integrity and neuromodulation, which affect the transmission and integration of information between grey matter brain regions and brain function [41].

ADHD-related atypical white matter structural integrity refers to differences in fibre density, axonal diameter and myelination. These differences are reflected in increased and decreased fractional anisotropy results. For example, hyperconnectivity (a larger than expected number of white matter tract fibres) or hypoconnectivity (a smaller than expected number of white matter tract fibres) findings [24, 42, 47]; significantly reduced P3 amplitude [48]; and significantly higher-than-normal N-acetylaspartate levels, a marker of neuronal activity in children [49]. In comparison to neurotypical peers, individuals with ADHD may display microstructural and connectivity differences in the commissural fibres that connect the bilateral brain hemispheres (i.e., corpus collosum), association fibres that link adjacent gyri or more distant cortical brain regions (i.e., uncinate fasciculus, superior and inferior longitudinal fasciculus, cingulum, inferior fronto-occipital fasciculus), and projection fibres that connect cortical brain regions with the brain stem (i.e., corona radiata) [36, 50, 53]. Similarly, individuals with ADHD may display microstructural and connectivity differences in large-scale brain networks. This includes the default mode network; cognitive control (frontoparietal) network which facilities executive control and goal attainment; ventral attention network which is implicated in maintaining attentional arousal and alertness; dorsal attention network which oversees selective and sustained attention; salience network which is implicated in reward and emotional based decision-making; and the limbic/affective network which governs emotional regulation and social cognition [32, 36, 40, 54, 58].

Of note when interpreting these findings, spatial convergence of ADHD-related hyperconnectivity or hypoconnectivity has not been achieved [59] except in a significant cluster in the left superior temporal gyrus [60]. Furthermore, electrophysiology studies report highly heterogenous and measurement dependent alterations in theta/beta ratio, which is a marker of inattention [61, 63], as well as atypical event-related potentials during tasks involving inhibitory control, attention, working memory, and performance monitoring [64]. There is no evidence that ADHD is associated with altered resting-state vagal tone [65], and the evidence for autonomic nervous system modulation and elevated theta/beta ratio in ADHD is low and subject to significant clinical and methodological heterogeneity [66, 69].

Regarding neuromodulation, many of the ADHD-associated brain regions that may be reduced in volume, such as the cerebellar vermis [70], and all of the large-scale functional networks that tend to be atypical in this cohort, have activities that are modulated by the monoamines dopamine and noradrenaline [71]. This includes the salience and limbic networks that encompass the mesolimbic dopamine pathway that mediates reward processing and motivation [58, 72], and the mesocortical pathway that mediates inhibitory control, timing and attention regulation [73]. Monoamine modulation is thought to be regulated by genetic factors, with serotonin and dopamine transporter and receptor genes having been implicated in ADHD [70, 73]. Further evidence of ADHD-related atypical neuromodulation includes stimulant medications used to treat ADHD target dopaminergic and noradrenergic modulation [71], and in-the-moment cognitive performance in individuals with ADHD appears to improve in response to reinforcement, which theoretically stimulates phasic dopamine release [74]. Additionally, it is possible that children and adults with ADHD, in comparison to neurotypical peers, display small differences in dopamine transporter levels. However, evidence for atypical dopamine transporter levels is weak [73], and results are compounded by factors that make definitive conclusions difficult i.e., prior exposure to long-term stimulant medication treatment and smoking [73]. Dopamine transporter binding is also likely influenced by complex interactions between other receptors and monoamines [73]. Regarding cortisol, research has also examined the relationship between this neuromodulator and ADHD, but no association has been found [75].

### Atypical Cognitive Abilities

The findings of 70 papers included in Table 1 converge to indicate that ADHD is associated with heterogeneous differences in executive (e.g., working memory, inhibitory control, cognitive flexibility) and non-executive (e.g., alerting attention, reward processing, long-term memory) cognitive processes. Reaction time and processing speed along with time perception and estimation, planning, and complex decision making/problem-solving, also appear to be impacted by ADHD.

### Working Memory

The findings of 22 papers (see Supplementary information, Appendix J) indicate that in comparison to neurotypical peers, individuals with ADHD may exhibit poorer overall auditory [76], visual and verbal (phonological) working memory performance [77, 78]. They may also display atypical activation within the prefrontal cortex during tasks reliant on working memory [39, 79, 80].

The magnitude of the working memory problems experienced by children and adults with ADHD appears to increase in line with (1) task complexity or demand, and/or (2) the number of consecutive tasks reliant on working memory performance [77, 78]. Alderson et al. [77] report that tasks reliant on memory recall were associated with larger between-group differences than recognition tasks, likely because recall tasks place greater demand on effortful cognitive processes. Alderson et al. [77] and Kasper et al. [78] report a positive relationship between the number of experimental working memory tasks incorporated into a study and the working memory problems displayed by individuals with ADHD, likely because working memory demands have a cumulative effect on performance.

A child’s ability to store, maintain and manipulate pertinent information in working memory is influenced by their ability to screen out irrelevant or distracting external sensory stimuli along with unwanted thoughts and memories [81]. Therefore, the working memory problems children with ADHD may experience are likely compounded by ADHD-related inhibitory control challenges [81].

### Inhibitory Control

Relatedly, the findings of 56 papers (see Supplementary information, Appendix K) indicate children and adults with ADHD, in comparison to their typically developing peers, tend to exhibit moderately more problems with inhibitory control. These can be in the form of inefficient (1) response (motor/behaviour) inhibition [82, 83] and/or (2) interference control challenges [84]. They also display (3) atypical activation within the prefrontal cortex, fronto-parietal network and cortioco-striato-thalamo-cortical loops during inhibition tasks [55, 58, 79, 85, 87], and (4) reduced basal ganglia, thalamus, and cerebellum grey matter volumes, which have been linked to poor impulse control and attention regulation [88].

Regarding atypical response inhibition, children and adults with ADHD are prone to ocular inhibition errors, although results are highly heterogenous [89], excessive body movement (hyperactivity), rapid responses, risky decision-making [90, 93], and emotional impulsivity/ dysregulation [94]. They tend to make more commission errors (i.e., respond when no target is presented) during neurocognitive tests [95] and to display attenuated error negativity or problems with error monitoring [96]. The number of errors they make may disproportionately increase in situations that demand a faster event response as the pre-potency of the Go response is heightened making ‘braking’ to No-go stimuli harder [95]. Furthermore, children and adults with ADHD may display diminished post-error slowing [97]. That is, rather than slow down post error, they tend to sustain or even increase their response speed [97].

In comparison to healthy developing peers, children and adolescents with ADHD are also likely to exhibit difficulty delaying gratification, instead choosing smaller, immediate rewards over larger delayed rewards as they tend to discount the value of a reward the longer it is delayed [92, 98, 99]. In the presence of reinforcement, response inhibition performance in children and adolescents with ADHD may however normalise depending upon the reinforcement schedule and the type of rewards on offer, making reinforcement a useful strategy for supporting inhibitory control [100, 101]. Although the magnitude of the behaviour inhibition challenges children and adolescents with ADHD display appear to decrease with age, many individuals with ADHD continue to display poor response inhibition in adulthood [102].

Regarding atypical interference control, children and adolescents with ADHD tend to exhibit poor attention inhibition, which may extend into adulthood [103]. This can be in the form of problems ignoring irrelevant or distracting external sensory stimuli [94] and atypical attention interference control speed as indexed by reaction time and accuracy [104]. Cognitive attention inhibition may also be affected as individuals with ADHD display greater levels of spontaneous, unintentional mind wandering [105], which appears to disrupt self-directed or self-referential cognition and impact goal attainment [58, 72], possibly due to a lack of correlation between the cognitive control and default mode networks [58]. While it is possible visual search accuracy is additionally compromised in the presence of ADHD, the evidence for inaccurate performance problems during visual search tasks is inconclusive [106], but appears to be more compromised in less or more complex serial search tasks [107].

### Cognitive Flexibility

The findings of 9 papers (See Supplementary information, Appendix L) suggest that children and adults with ADHD tend to display less cognitive flexibility than their neurotypical peers. This is reflected in greater difficulty with cognitive fluency (information processing) [108], task switching (set switching) [55, 109, 110], and atypical activation within the prefrontal cortex during tests measuring cognitive flexibility [80].

### Alerting Attention and Attentional Vigilance

The findings of 23 papers (see Supplementary information, Appendix M) indicate, in comparison to healthy peers, children and adults with ADHD tend to experience problems achieving and sustaining optimal alertness (arousal) and maintaining attentional vigilance (the ability to detect a rare critical event) during prolonged activities [94, 103, 111]. As a result they may display (1) decreased perceptual sensitivity or poorer signal detection during continuous tasks, (2) slower drift rates and more variable and slower reaction times when performing tasks related to sustained attention [95, 112], as well as (3) commit more omission errors (i.e., fail to respond to a stimuli) [112]. The underlying cause may be variously attributed to children and adults with ADHD displaying hypoconnectivity between the regions of the ventral attention network [55, 113], possible impairments in the alerting and executive networks [114], excessive daytime sleepiness [115], and atypical autonomic nervous system arousal during resting times and during tasks requiring sustained attention (i.e., when processing cognitive, rewarding or socio-emotional information) [116].

### Reward Processing

The findings of 13 papers (see Supplementary information, Appendix N) indicate children and adults with ADHD may display atypical reward processing. This is indexed by possible ventral-striatal [74] and caudate hypo-responsiveness during reward anticipation [31]; significant differences in reward evaluation and reward utility predictions; delay aversion and preferences for immediate rewards over larger delayed rewards; heightened sensitivity to rewards; and increased temporal discounting (i.e., the value of a reward loses its salience when achievement is delayed) [98, 99, 98–99]. Furthermore, rewards may ameliorate response inhibition problems in children and adults with ADHD more than in controls [100]. They may also normalise performance to the baseline level of controls [100].

### Long-Term Memory

The findings of 11 studies (see Supplementary information, Appendix O) indicate that in comparison to neurotypical peers, adults with ADHD potentially display atypical semantic memory (verbal long-term memory acquisition and recognition/verbal fluency of facts, ideas and concepts) when presented with verbal cues [109, 119]. This may be due to atypical semantic memory interference control, which affects memory acquisition and delayed recall performance [120, 121].

When presented with a visual picture, evidence for ADHD-related long-term episodic memory performance (or visual fluency) is equivocal [108, 121]. However, two forms of long-term memory that rely on both declarative memory components, semantic and episodic memory, are possibly implicated in ADHD; prospective and associative memory. Individuals with ADHD potentially display atypical time-based and event-based prospective memory (or remembering to carry out an action at an appropriate future moment), which makes them prone to response errors and compromised performance [122]. Additionally, during more complex and more life-like conditions (e.g., in the presence of distractions, or when there is a delay between the discriminative event and the response), individuals with ADHD possibly display atypical associative memory [123]. The associative memory difficulties children with ADHD display are in the form of aberrant conditional discrimination learning and reversal learning challenges, both of which likely impede learning via operant conditioning during real world situations [123].

In contrast, procedural learning, a component of non-declarative memory, appears to be in tack [124].

ADHD-related long-term memory difficulties are postulated to be due to learning deficits induced at the stage of memory encoding, and likely reflect the underlying working memory, inhibitory control (response inhibition and attentional interference control) and cognitive flexibility differences associated with ADHD [121].

### Reaction Time and Processing Speed

Thirteen studies (see Supplementary information, Appendix P) provide evidence that ADHD is associated with atypical reaction times [110, 125], considerable reaction time variability [126, 129], and atypical processing speed [94, 103, 130]; although processing speed may be unaffected when reaction time variability is accounted for [129]. Evidence of atypical reaction time and reaction time variability include individuals with ADHD appearing to acquire information and reacting more slowly during slow event rate tasks, as reflected in displays of decreased perceptual sensitivity and slower attentional drift rates, processing speed, and reaction times [95, 112]. However, during faster event rate tasks or post error they tend to display faster reaction times [97, 131]. Of note, is that it is unclear whether reaction time differences exist during tasks involving emotional facial expressions [132].

### Time Perception/Estimation, Planning and Complex Decision-Making/Problem-Solving

Fourteen studies (see Supplementary information, Appendix Q) provide evidence that ADHD is associated with differences in time perception, planning and complex decision-making/problem-solving. Individuals with ADHD possibly display less accurate and less precise time perception and a higher tendency to overestimate time, although the number of studies examining time perception in ADHD is scarce [133, 135]. Activation in typical brain areas related to timing may also be significantly reduced in people with ADHD [136]. In regards to planning [110, 111, 137] and decision-making, reward-based decision-making [138] along with abstract and visual/figural problem-solving and reasoning, appear to be most significantly impacted in the presence of ADHD [108].

## Discussion

This integrative review synthesised evidence on the impact of ADHD on the brain and cognitive function in order to gain greater insight into the biologically-based, innate person characteristics that influence a child and adult with ADHD’s functional capacity.

The findings of the review suggest ADHD is possibly associated with a significant delay in cortical maturation. As brain maturation generally follows a predictable developmental trajectory that aligns with chronological age and results in (1) the differentiation of neural structures, (2) the emergence of cognitive processes, and (3) improvements in processing speed, the amount of information that can be mentally manipulated in working memory, and the complexity of such mental manipulations [139, 142], this delay in cortical maturation likely postpones cognitive maturation. It may also result in children with ADHD having cognitive and self-regulation skills that significantly lag behind their typically developing peers. This implies children with ADHD likely require supporting scaffolding that bridges the gap between their innate capacity and age-related expectations placed on them and to be successful.

The findings also indicate ADHD is also associated with anatomical, functional and cognitive differences, many of which tend to remain in adulthood. Individuals with ADHD may display atypical connectivity within and between various regions of the brain that likely contribute to differences in cognitive processing [44]. Both commissural fibres and association fibres appear to be implicated, along with many large-scale brain networks. They may also display heterogeneous differences in executive and non-executive abilities that can impact higher order cognitive processing. These include working memory, inhibitory control, cognitive flexibility, alerting attention, reward processing, and long-term memory. The cognitive processes related to reaction time, time perception and estimation, planning and complex decision-making/problem-solving also appear to be heterogeneously impacted. Furthermore, in-the-moment cognitive performance in individuals with ADHD may improve in response to scaffolding in the form of reinforcement [74], while reward delay may impose a demotivational influence [99].

The anatomical, functional and cognitive differences associated with ADHD likely influence an individual’s ability to maintain alertness (arousal), attentional vigilance and focus; stop (inhibit a habitual/instinctual response), evaluate choice alternatives and make informed decisions before responding with words or actions; develop self and social awareness; listen and learn; perceive time; plan, prioritise, stay organised and take action over time; successfully achieve their goals; and navigate social expectations. Of note is that these challenges are neuropsychological in nature. They are not a choice, and outside of an individual with ADHD’s ability to control [7]. The cognitive differences associated with ADHD also have no diagnostic utility due to the heterogeneous nature of the study findings [14], and because intelligence has been shown to significantly mediate cognitive tests results in the presence of ADHD [143].

## Implications

Together, the findings of this paper highlight the need for clinicians to ensure healthcare interventions provided to children and adults with ADHD align with their neurocognitive profile. One way this can be accomplished is by including this information in education interventions in a way that is neuro-affirming; supports the development of self-understanding, self-acceptance, self-compassion; and facilitates positive adaption and personal empowerment via the use of supporting scaffolding. This information is vital for ensuring parents and teachers are able to (1) sensitively understand and interpret a child’s ADHD-related symptoms and behavioural tendencies, (2) appreciate what they are likely to be able to do or not do at a particular age, (3) proactively ensure there is a good fit to their inherited vulnerabilities, and either the demands placed upon them or the scaffolding and support provided, and (4) commence the process of collaboratively fostering in them protective and recovery-oriented traits so that they reach adulthood with their self-esteem intact and have the knowledge and skills required to navigate life successfully in adulthood. For adults with ADHD this information can help combat the shame they have often internalised due to life-long negative messages and experiences of failure, as well as provide insight into the type of scaffolding they can put in place to support their ADHD-related cognitive differences so that they can set themselves up for success and protect themselves from disappointment and harm. We aim to use these findings to develop a ‘framework/checklist” that parents, adults and clinicians can use to identify the possible mechanisms that may be contributing to an individual with ADHD’s challenges and the scaffolding that may help to facilitate hope, success, safety and personal empowerment. We also wanted to ensure this information is easily accessible so it can be used to develop education interventions that support ADHD literacy and the identification of appropriate healthcare interventions. We acknowledge that the findings, however, will need to be tailored to meet the needs of different recipient groups in order for them to be comprehensible and meaningful.

More specifically, understanding the underlying reasons why individuals with ADHD display difficulties with response inhibition for example, provides healthcare providers, educators and individuals with ADHD and their parents insight into why it can be almost impossible for individuals with ADHD to ‘stop and think before speaking or acting’ without medication that increases synaptic dopamine availability and scaffolding that assists an individual to delay (i.e., access barriers) or prevents a response (i.e., removal of temptations from sight). Acknowledging individuals with ADHD can lack self-awareness due to problems with interference control [58, 72], which impacts self-monitoring and self-exploration [144], explains the individuals with ADHD require support that gently facilitates introspective attention and the development of self-awareness. Understanding ADHD-related visual working memory clarifies why it is important for individuals with ADHD to make things visible, while problems with prospective memory illustrates why setting alarms and using visual prompts is very helpful. While understanding (1) how rewards can induce phasic dopamine release and in doing so support cognitive function depending upon their perceived valence, and (2) reward delay can have a demotivating influence on individuals with ADHD due to delay discounting, provides insight into the need for positive reinforcement to be in-the-moment and commensurate with the values of an individual with ADHD. However, as reinforcement is unable to change an underlying developmental delay [7], expectations of what reinforcement can achieve also needs to align with an individual’s cognitive capacity, otherwise a reward can easily become a form of punishment.

Clinicians, teachers and parents should also consider the impact ADHD likely has on all forms of learning as well as all aspects of the emotional regulation process when providing guidance on support interventions to ensure they align with the neurocognitive profile associated with ADHD. For example, ADHD-related attention regulation difficulties likely disrupt observational learning and classical conditioning, as well as the ability to employ distractions techniques (attentional deployment) as a means of regulating emotions. ADHD-inhibition and reward processing differences likely impact learning via operant conditioning as well as the ability to stop, think and practice situation selection and situation modification in order to avoid being emotionally triggered.

## Limitations

Several limitations should be taken into account when considering the findings of this integrative review. Firstly, significant levels of heterogeneity were found in many of the included review papers. This heterogeneity likely reflects differences in the examined hypotheses, assessed clinical populations, inclusion and exclusion criteria including whether medication usage or co-morbidity was taken into consideration, experimental design (we did not examine the impact different brain imaging techniques had on findings), meta-analytic methods, calibration across scanners, applied statistical thresholds, data-analysis approaches, and reporting practices [24, 59, 72, 116]. Furthermore, the findings of many included studies may be influenced by the presence of comorbidity as some captured studies included primary studies that (1) didn’t report on comorbidity or (2) included participants with comorbidity, while others did not address the potential influence identified comorbidity had on study findings via meta-regression. A dual diagnosis of autism and ADHD was also not permitted until the release of the Diagnostic Statistical Manual of Mental Disorders (DSM), 5th edition, published in 2013. Whether this is an issue remains unclear. While some researchers argue that comorbid disorders “should always be statistically controlled to ensure that the neuropsychologic impairments associated with ADHD cannot be explained more parsimoniously” (Lahey et al., 1998, as cited in [83], p. 1342), others researchers argue that as the majority of individuals with ADHD have a comorbid disorder, restricting a sample to those without comorbidities would severely reduce the generalisability of study findings [58].

Although recently reported effect sizes have become more reliable and reported heterogeneity rates have reduced with increased study rigor [77, 81], it is likely ADHD research findings will always remain somewhat heterogeneous due to both the equifinality (different originating risks leading to the same clinical outcome) and the multi-finality (the same pattern of risk leading to a different outcome) associated with ADHD [145]. That is, ADHD heterogeneity will continue to reflect the development of divergent, individual ontological phenotypes due to interactions between an ADHD individual’s genetic predisposition and social environmental exposure [14, 15], and their ability to adapt or use compensatory strategies (Frazier et al., 2007 cited in [77]).

Secondly, the number of studies included in captured reviews focusing on a particular phenomenon likely impacted findings. Gao et al. [55] reported that the number of studies examining differences in the individual large-scale brain networks of people with ADHD was relatively small, which made integrating the results for the VAN, DAN, SSN and visual network difficult via meta-analysis. Viera de Melo et al. (2018) reported studies providing aggregated results of fMRI reviews often included trials with functional tasks that were not fully described. This made it impossible for them to identify the different methods, variations and individual contribution each task made to the final effect sizes [24].

Thirdly, although the neurocognitive differences associated with ADHD also contribute to the strengths that individuals with the condition display, no captured studies interpreted findings in a positive light or provided insight into the positive traits that can accompany ADHD. For example, while research has identified that ADHD is associated with difficulties such as set-shifting and task-switching, these difficulties also likely contribute to ADHD-related hyperfocus, which is often viewed as being a strength-based trait by individuals with the condition as it supports high productivity and flow [146], even though hyperfocus can also be problematic. Individuals with ADHD also report that although ADHD-related impulsivity can lead to poor outcomes, it also likely contributes to their adventurous, spontaneous nature and makes life fun [146]. Additionally, ADHD-related mind wandering which interrupts the focus and concentration, may underlie ADHD-related traits such as high creativity [147], divergent thinking, uninhibited imagination [148], and ability to think outside of the box [146]. As narratives and discourse around ADHD influences how people with the condition view and understand themselves, identifying ADHD-related strengths that individuals with the condition can recruit in order to function at a high level [149], is vital for reducing the stigma surrounding ADHD. It could assist individuals with the condition to identify scaffolding they could put in place to support their cognitive differences as well as facilitate hope, perception of competence, resilience, adaption to disability and psychological well-being [150].

## Conclusion

ADHD tends to affect a person across the life course. This review found ADHD is associated with (1) a significant delay in cortical maturation along with differences in neuro anatomy that do not appear to fully resolve in adulthood, (2) atypical brain and (3) atypical cognitive processes. The cognitive functions heterogeneously implicated by the presence of ADHD include working memory, inhibitory control, cognitive flexibility, alerting attention, reward processing, and long-term memory, along with reaction time, time perception and estimation, planning and complex decision making/problem-solving.

## Supplementary Information

Below is the link to the electronic supplementary material.

Supplementary file1 (PDF 438 KB)

## Data Availability

No datasets were generated or analysed during the current study.
