# Supplementary material for: Increasing Health Literacy on ADHD: A Cross-Disciplinary Integrative Review Examining the Impact of ADHD on Brain Maturation, Composition and Function and Cognitive Processes Across the Life Course
Source: Child Psychiatry Hum Dev. 2025 Feb 26;57(6):1834–72. doi: 10.1007/s10578-025-01815-5 (PMC13748260; doi:10.1007/s10578-025-01815-5)
Supplement: Supplementary file 1 [file 10578_2025_1815_MOESM1_ESM.pdf]

Increasing health literacy on ADHD: A cross-disciplinary integrative review examining the impact of ADHD on brain maturation, composition, and connectivity and cognitive function across the life course

Appendix A
Inclusion/exclusion criteria

Inclusion: Review studies and longitudinal neuroimaging case-control studies were included if they reported on the influence of ADHD on brain maturation, composition (anatomy and morphology), connectivity, cognitive development, or cognitive function. Reviews examining other conditions along with ADHD (e.g., autism) were included in the integrative review so long as they compared participants with ADHD against healthy developing peers.

Exclusion: Individual empirical studies including clinical trials and randomised control trials, non-structured literature reviews (i.e., narrative reviews), and publications that could not be identified using a research database (i.e., education articles and grey literature), were excluded. Other studies excluded included: genomics studies; studies using animal models; studies examining personality traits, intelligence quotient, language or learning disorders; and studies or papers *primarily* focused on: providing a general overview of all ADHD; highlighting the role of a neuroimaging method or discussing methodological advances and limitations; non-neuromodulator hormone contribution to ADHD other than BNDF; the workings of pharmacological agents; the implications for treatment interventions; and model or theory proposal, development or review.

Appendix B: Quality assessment tools

An adapted version of the JBI Critical Appraisal Checklist for systematic reviews and research syntheses (Aromataris et al., 2015) was used to screen the quality of the meta-analysis and systematic, structured, integrative and scoping review studies.

**Reviewer**		**Date**		
**1st author**		**Year**		
**Criteria**		**Yes**	**No**	**U/C**
1	Is the review question and/or aim clearly stated?			
2	Was the inclusion criteria clearly defined/ appropriate?			
3	Was the search strategy appropriate?			
4	Was the criteria for appraising studies (quality assessment) appropriate?			
5	Did two authors independently screen papers for eligibility?			
6	Were efforts made to minimise errors in data extraction i.e., extraction conducted by two or more reviewers independently?			
7	Was the likelihood of publication bias statistically assessed?			
8	Was appropriate statistical analysis applied to combine studies?			
9	Was the impact of comorbidity (confounding factor) determined statistically?			
Quality score: 0 1 2 3 4 5 6 7				
Comment:				

An adapted version of the AXIS Critical Appraisal of Cross-Sectional Studies (Downes et al., 2016) was used to assess the quality of the longitudinal and large cohort neuroimaging case-control studies captured by hand search.

**Reviewer**	Louise Brown	**Date**	22/01/24	
**1st author**		**Year**		
**Criteria**		**Yes**	**No**	**U/C**
1	Was the aim/objective of the study clear			
2	Was the sample size justified?			
3	Was the target/reference population clearly defined?			
4	Was the sample base taken from an appropriate population?			
5	Were measures undertaken to address and categorise non-responders?			
6	Was the measurement tool appropriate to the aim of the study?			
7	Were the methods clearly explained?			
8	Was the impact of comorbidity (confounding factor) determined statistically?			
Quality score: 0 1 2 3 4 5 6 7				
Comment:				

Quality score key:

0, 1 or 2 = high quality, 3 or 4 = moderate quality, 5 or 5 = low quality

Appendix C
Screened studies that did not meet the inclusion criteria

Non-structure reviews (narrative)

Baroni, A., & Castellanos, F. X. (2015). Neuroanatomic and cognitive abnormalities in attention-deficit/hyperactivity disorder in the era of 'high definition' neuroimaging. *Current Opinion in Neurobiology*, *30*, 1-8. https://doi.org/10.1016/j.conb.2014.08.005

Cao, M., Shu, N., Cao, Q., Wang, Y., & He, Y. (2014). Imaging functional and structural brain connectomics in attention-deficit/hyperactivity disorder. *Molecular Neurobiology*, *50*(3), 1111-1123. https://doi.org/10.1007/s12035-014-8685-x

Cherkasova, M. V., & Hechtman, L. (2009). Neuroimaging in attention-deficit hyperactivity disorder: Beyond the frontostriatal circuitry. *Canadian Journal of Psychiatry*, *54*(10), 651-664. https://doi.org/10.1177/070674370905401002

Cook, N. E., Braaten, E. B., & Surman, C. B. H. (2018). Clinical and functional correlates of processing speed in pediatric attention-deficit/hyperactivity disorder: A systematic review and meta-analysis. *Child Neuropsychology*, *24*(5), 598-616. https://doi.org/10.1080/09297049.2017.1307952

Cortese, S. (2012). The neurobiology and genetics of attention-deficit/hyperactivity disorder (ADHD): What every clinician should know. *European Journal of Paediatric Neurology*, *16*(5), 422-433. https://doi.org/10.1016/j.ejpn.2012.01.009

di Michele, F., Prichep, L., John, E. R., & Chabot, R. J. (2005). The neurophysiology of attention-deficit/hyperactivity disorder. *International Journal of Psychophysiology*, *58*(1), 81-93. https://doi.org/10.1016/j.ijpsycho.2005.03.011

Dougherty, C. C., Evans, D. W., Myers, S. M., Moore, G. J., & Michael, A. M. (2016). A comparison of structural brain imaging findings in autism spectrum disorder and attention-deficit hyperactivity Disorder. *Neuropsychology Review*, *26*(1), 25-43. https://dx.doi.org/10.1007/s11065-015-9300-2

Doyle, A. E. (2006). Executive functions in attention-deficit/hyperactivity disorder. *Journal of Clinical Psychiatry*, *67 Suppl 8*, 21-26. https://www.psychiatrist.com/wp-content/uploads/2021/02/24673_executive-functions-attention-deficit-hyperactivity.pdf

Faraone, S. V., Biederman, J., & Mick, E. (2006). The age-dependent decline of attention deficit hyperactivity disorder: A meta-analysis of follow-up studies. *Psychological Medicine*, *36*(2), 159-165. https://doi.org/10.1017/s003329170500471x

Gharehgazlou, A., Freitas, C., Ameis, S. H., Taylor, M. J., Lerch, J. P., Radua, J., & Anagnostou, E. (2021). Cortical gyrification morphology in individuals with ASD and ADHD across the lifespan: A systematic review and meta-analysis. *Cerebral Cortex*, *31*(5), 2653-2669. https://doi.org/10.1093/cercor/bhaa381

Jadidian, A., Hurley, R. A., & Taber, K. H. (2015). Neurobiology of adult ADHD: Emerging evidence for network dysfunctions. *Journal of Neuropsychiatry & Clinical Neurosciences*, *27*(3), 173-178. https://doi.org/10.1176/appi.neuropsych.15060142

Krause, K. H., Dresel, S. H., Krause, J., la Fougere, C., & Ackenheil, M. (2003). The dopamine transporter and neuroimaging in attention deficit hyperactivity disorder. *Neuroscience & Biobehavioral Reviews*, *27*(7), 605-613. https://doi.org/10.1016/j.neubiorev.2003.08.012

Lenartowicz, A., Mazaheri, A., Jensen, O., & Loo, S. K. (2018). Aberrant modulation of brain oscillatory activity and attentional impairment in attention-deficit/hyperactivity disorder. *Biological Psychiatry: Cognitive Neuroscience & Neuroimaging*, *3*(1), 19-29. https://doi.org/10.1016/j.bpsc.2017.09.009

Liston, C., Malter Cohen, M., Teslovich, T., Levenson, D., & Casey, B. J. (2011). Atypical prefrontal connectivity in attention-deficit/hyperactivity disorder: Pathway to disease or pathological end point? *Biological Psychiatry*, *69*(12), 1168-1177. https://doi.org/10.1016/j.biopsych.2011.03.022

Luman, M., Oosterlaan, J., & Sergeant, J. A. (2005). The impact of reinforcement contingencies on AD/HD: A review and theoretical appraisal. *Clinical Psychology Review*, *25*(2), 183-213. https://doi.org/10.1016/j.cpr.2004.11.001

Martinussen, R., & Tannock, R. (2006). Working memory impairments in children with attention-deficit hyperactivity disorder with and without comorbid language learning disorders. *Journal of Clinical and Experimental Neuropsychology*, *28*(7), 1073-1094. https://doi.org/10.1080/13803390500205700

Mueller, A., Hong, D. S., Shepard, S., & Moore, T. (2017). Linking ADHD to the neural circuitry of attention. *Trends in Cognitive Sciences*, *21*(6), 474-488. https://doi.org/10.1016/j.tics.2017.03.009

Noreika, V., Falter, C. M., & Rubia, K. (2013). Timing deficits in attention-deficit/hyperactivity disorder (ADHD): Evidence from neurocognitive and neuroimaging studies. *Neuropsychologia*, *51*(2), 235-266. https://doi.org/10.1016/j.neuropsychologia.2012.09.036

Perlov, E., Philipsen, A., Matthies, S., Drieling, T., Maier, S., Bubl, E., Hesslinger, B., Buechert, M., Henning, J., Ebert, D., & Tebartz Van Elst, L. (2009). Spectroscopic findings in attention-deficit/hyperactivity disorder: Review and meta-analysis. *World Journal of Biological Psychiatry*, *10*(4 Pt 2), 355-365. https://doi.org/10.1080/15622970802176032

Rubia, K., Alegria, A., & Brinson, H. (2014). Imaging the ADHD brain: Disorder-specificity, medication effects and clinical translation. *Expert Review of Neurotherapeutics*, *14*(5), 519-538. https://doi.org/10.1586/14737175.2014.907526

Rubia, K., Alegria, A. A., & Brinson, H. (2014b). Brain abnormalities in attention-deficit hyperactivity disorder: A review. *Revista de Neurologia*, *58 Suppl 1*, S3-16.

Shiels, K., & Hawk, L. W., Jr. (2010). Self-regulation in ADHD: The role of error processing. *Clinical Psychology Review*, *30*(8), 951-961. https://doi.org/10.1016/j.cpr.2010.06.010

Tamm, L., Narad, M. E., Antonini, T. N., O'Brien, K. M., Hawk, L. W., Jr., & Epstein, J. N. (2012). Reaction time variability in ADHD: A review. *Neurotherapeutics*, *9*(3), 500-508. https://doi.org/10.1007/s13311-012-0138-5

Tsai, S. J. (2017). Role of neurotrophic factors in attention deficit hyperactivity disorder. *Cytokine & Growth Factor Reviews*, *34*, 35-41. https://doi.org/10.1016/j.cytogfr.2016.11.003

Van Mourik, R., Oosterlaan, J., & Sergeant, J. A. (2005). The Stroop revisited: A meta-analysis of interference control in AD/HD. *Journal of Child Psychology & Psychiatry*, *46*(2), 150-165. https://doi.org/10.1111/j.1469-7610.2004.00345.x

Weyandt, L. L. (2005). Executive function in children, adolescents, and adults with attention deficit hyperactivity disorder: Introduction to the special issue. *Developmental Neuropsychology*, *27*(1), 1-10. https://doi.org/10.1207/s15326942dn2701_1

Woods, S. P., Lovejoy, D. W., & Ball, J. D. (2002). Neuropsychological characteristics of adults with ADHD: A comprehensive review of initial studies. *Clinical Neuropsychologist*, *16*(1), 12-34. https://doi.org/10.1076/clin.16.1.12.8336

Zhu, Y., Yang, D., Ji, W., Huang, T., Xue, L., Jiang, X., Chen, L., & Wang, F. (2016). The relationship between neurocircuitry dysfunctions and attention deficit hyperactivity disorder: A review. *BioMed Research International*, *2016*, 3821579. https://doi.org/10.1155/2016/3821579

Zhu, Y., Jiang, X., & Ji, W. (2018). The mechanism of cortico-striato-thalamo-cortical neurocircuitry in response inhibition and emotional responding in attention deficit hyperactivity disorder with comorbid disruptive behavior disorder. *Neuroscience Bulletin*, *34*(3), 566-572. https://doi.org/doi.org/10.1007/s12264-018-0214-x

Structured review

Gosse, L. K., Bell, S. W., & Hadi, S. M. (2022). Functional near-infrared spectroscopy in developmental psychiatry: A review of attention deficit hyperactivity disorder. *European Archives of Psychiatry & Clinical Neuroscience, 272*(2), 273-290.

Harikumar, A., Evans, D. W., Dougherty, C. C., Carpenter, K. L. H., & Michael, A. M. (2021). A Review of the Default Mode Network in Autism Spectrum Disorders and Attention Deficit Hyperactivity Disorder. *Brain Connectivity, 11*(4), 253-263.

Single studies

Yordanova, J., Kolev, V., & Rothenberger, A. (2013). Event-related oscillations reflect functional asymmetry in children with attention deficit/hyperactivity disorder. *Supplements to Clinical Neurophysiology*, *62*, 289-301. https://doi.org/10.1016/b978-0-7020-5307-8.00018-1

Appendix D
Composite summary of allocated quality scores

**Review and meta-analysis studies**		**n = 105**			
**Criteria**		**Yes**	**No**	**UC**	**N/A**
1	Is the review question and/or aim clearly stated?	105	-	-	-
2	Was the inclusion criteria clearly defined / appropriate?	97	2	6	-
3	Was the search strategy appropriate?	101	-	4	-
4	Was the criteria for appraising studies (quality assessment) appropriate?	16	89	-	-
5	Did two authors independently screen papers for eligibility?	42	2	61	-
6	Were efforts made to minimise errors in data extraction i.e., extraction conducted by two or more reviewers independently?	31	1	73	-
7	Was the likelihood of publication bias statistically assessed?	42	62	-	*1
8	Was appropriate statistical analysis applied to combine studies?	81	24	-	-
9	Was the impact of comorbidity (confounding factor) determined statistically?	14	74	1	**16
**Quality Score:** 0 **(n=3)** 1 **(n=4)** 2 **(n=16)** 3 **(n=19)** 4 **(n=27)** 5 **(n=20)** 6 **(n=11)** 7 **(n=5)**					
**Ranking**	High: n = 23 Moderate: n = 46 Low: n = 31 Very low: n= 5				

**Longitudinal and large cohort neuroimaging case-control studies**		**n = 9**			
**Criteria**		**Yes**	**No**	**U/C**	**N/A**
1	Was the aim/objective of the study clear	9	-	-	-
2	Was the sample size justified?	9	-	-	-
3	Was the target/reference population clearly defined?	9	-	-	-
4	Was the sample base taken from an appropriate population?	9	-	-	-
5	Were measures undertaken to address and categorise non-responders?	3	1	2	***3
6	Was the measurement tool appropriate to the aim of the study?	9	-	-	-
7	Were the methods clearly explained?	9	-	-	-
8	Was the impact of comorbidity (confounding factor) prevented or addressed?	3	3	-	**3
**Quality score:** 1 **(n = 9)** 2 3 4					
**Ranking**	High: n = 9 Moderate Low				

* Not applicable because paper was a review of meta-analysis.
**Not applicable because participants with comorbidities were excluded, therefore, need to determine impact mitigated.
***Not-applicable as ENIGMA data utilised.

Appendix E
Methods employed by the neuroimaging studies captured in this integrative review

Author	Date	MRI	f-MRI	RS-fMRI	t-fMRI	VMB	ERP	Blood test	TBSS	DTI	WB VBA	PET	ANS	MRS	EEG	HRV/ CVC	Other
Aoki	2018								*		*						
Aoki	2013																PMRS
Arns	2011														*		
Balogh	2016		*												*		Not stated
Boedhoe	2020	*															
Bellato	2020										*						
Boon	2000	*	*														
Chamorro	2022																OR
Chen	2023	*															
Chen	2016								*								
Cheng	2016						*										
Connaughton	2022	*															
Cortese	2021			*													
Cortese	2012		*														
Cui	2023								*								
Dickstein	2006		*									*					
Dutra	2016																TMS
Ellison-Wright	2008										*						
Frodl	2012					*											
Fusar-Poli	2012											*					SPECT
Gao	2019		*														
Geburek	2013																ERPs
Geiss	2023												*				
Hart	2013		*														
Hart	2012		*														
Hoogman	2019	*															
Hoogman	2017	*															
Hou	2023		*														
Hutchinson	2008	*															
Kaiser	2020						*										
Kamradt	2018							*									
Karalunas	2013																EEG
Koenig	2017															*	
Kowalczyk	2022		*														
Lei	2015		*														
Liu	2023		*														
Lukito	2020		*			*											
Ma	2016		*														

Mauri	2018																fNIRS
McCarthy	2015		*														
Michelini	2022																
Mills	2018	*															
Nakao	2011					*											
Paloyelis	2007		*														
Plitcha	2014		*														
Posner	2014			*													
Proal	2011					*											
Rash	2012															*	
Robe	2019															*	
Samea	2019		*		*	*											
Shaw	2013	*															
Shaw	2012	*															
Shaw	2007	*															
Snyder	2011														*		
Sutcubasi	2020			*													
Szuromi	2011						*										
Valera	2007	*															
van Ewijk	2012									*							
Vidor	2022													*			
Viera de Melo	2018	*	*			*			*	*				*			
Yap	2021				*												
Zhang	2023								*		*						
Zhang	2018							*									
Zhao	2022									*							

Key: MRI = magnetic resonance imaging, f-MRI = functional magnetic resonance imaging, RS-fMRI = resting state functional magnetic resonance imaging, t-fMRI = task functional magnetic resonance imaging, VBM = whole-brain voxel-based morphometry, ERP = event-related potential, BDNF = brain derived neurotrophic factor, TBSS = tract-based spatial statistics, DTI = diffusion tensor imaging, WBVBA – whole-brain voxel-based analysis, PET = positron emission tomography, ANS = autonomic nervous system, MRS = magnetic resonance spectroscopy, EEG = electroencephalogram, HRV = heart rate variability, CVC = cardiac vagal control, OR = oculomotor responses, TMS = transcranial magnetic stimulation, SPECT = single photon emission tomography
ERP = event rate potential, fNIRS = functional Near Infrared Spectroscopy.

Appendix F
Theme: Delayed Cortical Maturation and Atypical Neuroanatomy - ADHD and Atypical Cortical Maturation

First Author	Year	Quality	Findings – In Comparison to Controls
**Longitudinal Studies**			
Shaw	2012	High	For the R prefrontal cortex, the median age by which children with ADHD achieved peak thickness in 50% of cortical vertices was 14.6 years, significantly later than controls at 12.7 years. A similar but less pronounced delay in reaching cortical thickness was also found in the L hemispheric lobes.
Shaw	2007	High	Regional brain maturation progresses in a similar manner in both children with and without ADHD, with primary sensory areas attaining peak cortical thickness before polymodal, high-order association areas. However, children with ADHD attain peak cortical thickness approximately 3 years behind their typically developing peers.
**Review Studies**			
Frodl	2012	Mod	R globus pallidus and R putamen volumes are reduced in children with ADHD. These changes in the basal ganglia seem to diminish over time from child to adulthood.
Shaw	2006	High	Children with ADHD displayed global cortical thinning, most prominently in the medial and superior prefrontal and precentral regions, including the superior and medial frontal gyri and cingulate region bi-laterally, L precentral gyrus, and R anterior/mesial temporal cortex. Cortical thickness developmental trajectories did not differ significantly between participants with ADHD and control groups throughout except in the R parietal cortex, where trajectories converged by age 17.
**Large Cohort Studies**			
Boedhoe	2020	High	Children and adolescents with ADHD displayed reduced intercranial volume, reduced frontal surface area and striatal volume in comparison. None of the subcortical volumes differed significantly among adult patient groups.
Hoogman	2019	High	Children with ADHD, but not adolescents and adults with ADHD, displayed slightly reduced, but statistically significant, total cerebral cortex surface area. The most significant surface area reductions were noted in the superior frontal gyrus, lateral orbitofrontal cortex, posterior cingulate cortex, rostral anterior cingulate cortex and middle temporal gyrus. Children with ADHD, also displayed slightly reduced, but statistically significant, cortical thickness in 2 regions (fusiform gurus and temporal pole). These differences were most prominent in the group aged 10 and 11 years.
Hoogman	2017	High	Children with ADHD displayed small but significant reductions in intracranial volume in the nucleus accumbens, amygdala, caudate nucleus, hippocampus, and putamen. These differences were not present in adults with ADHD. Explorative lifespan modelling suggested a delay of maturation and a delay of degeneration.

Appendix G

Theme: Delayed Cortical Maturation and Atypical Neuroanatomy - ADHD and Atypical Neuroanatomy in Childhood

First Author	Year	Quality	Findings – In Comparison to Controls
**Review Studies**			
Chen	2023	High	Children with ADHD consistently displayed smaller amygdala surface area (particularly in the L hemisphere).
Boon	2020	Low	Individuals with ADHD display significant differences in neural anatomy (basal ganglia, thalamus, cerebellum, cerebral cortex grey matter volume, white matter tract disturbances, corpus callosum).
Lukito	2020	High	Children with ADHD display reductions in grey matter volume in ventromedial frontal cortex and subcallosal gyrus.
Aoki	2018	Mod	Children with ADHD display differences in fractional anisotropy values in a number of brain regions.
Viera de Melo	2018	Low	Children with ADHD display reduced overall cerebral volume, specifically, in the R hemisphere, frontal lobe, parietal lobe, basal nuclei [R caudate and putamen], R globus pallidus, corpus collosum, cerebellum, and vermis.
Frodl	2012	Mod	R globus pallidus and R putamen volumes are reduced in children with ADHD.
Fusar-Poli	2012	High	Children and adults with ADHD may display higher dopamine transporter density. Meta-regression analyses showed density was higher in patients with previous medication exposure and lower in medication-naive patients.
Nakao	2011	Low	Children with ADHD display (1) robustly localised global reductions in grey matter volume in the R lentiform nucleus (putamen & globus pallidus) extending to the caudate nucleus, and (2) slightly greater grey matter volume in the L posterior cingulate cortex.
Proal	2011	High	Children with ADHD displayed significantly decreased grey matter in the R caudate, R thalamus and bilateral cerebellar hemispheres.
Ellison-Wright	2008	Low	Children with ADHD displayed significant regional grey matter reduction the R putamen/globus pallidus region.
Hutchinson	2008	Mod	Female children and adolescents with ADHD displayed smaller splenium size. Male children and adolescents with ADHD display smaller rostral body size.
Valera	2007	Mod	Children with ADHD displayed heterogenous global volume reductions, the largest differences include cerebellar regions, the splenium of the corpus callosum, total and R cerebral volume, and R caudate.
**Longitudinal Studies**			
Hoogman	2019	High	Children with ADHD, but not adolescents and adults with ADHD, displayed slightly reduced, but statistically significant, total cerebral cortex surface area. The most significant surface area reductions were noted in the superior frontal gyrus, lateral orbitofrontal cortex, posterior cingulate cortex, rostral anterior cingulate cortex and middle temporal gyrus. Children with ADHD, also displayed slightly reduced, but statistically significant, cortical thickness in 2 regions (fusiform gurus and temporal pole).
Hoogman	2017	High	Children with ADHD displayed small but significant reductions in intracranial volume in the nucleus accumbens, amygdala, caudate nucleus, hippocampus, and putamen.
Shaw	2013	High	Adult ADHD status is associated with altered development and increased cortical thinning of components of the cortical networks and support attention, cognitive control, and the default mode network.
Shaw	2006	High	Children with ADHD displayed global cortical thinning, most prominently in the medial and superior prefrontal and precentral regions, including the superior and medial frontal gyri and cingulate region bi-laterally, L precentral gyrus, and R anterior/mesial temporal cortex. Cortical thickness developmental trajectories did not differ significantly between participants with ADHD and control groups throughout except in the R parietal cortex, where trajectories converged by age 17.
**Large Cohort**			
Boedhoe	2020	High	Children and adolescents with ADHD displayed reduced intercranial volume, reduced frontal surface area and striatal volume in comparison.

Appendix H
Theme: Delayed Cortical Maturation and Atypical Neuroanatomy - ADHD and Atypical Neuroanatomy in Adulthood

First Author	Year	Quality	Findings – In Comparison to Controls
**Review Studies**			
Chen	2023	High	Adults with ADHD consistently displayed smaller amygdala surface area (particularly in the L hemisphere).
Lukito	2020	High	The reductions in grey matter volume that children with ADHD display in the ventromedial frontal cortex and subcallosal gyrus do not fully resolve in adulthood.
Aoki	2018	Mod	Adults with ADHD display differences in fractional anisotropy values in a number of brain regions.
Frodl	2012	Mod	Adults with ADHD display reduced grey matter volumes in the anterior cingulate cortex.
Fusar-Poli	2012	High	Adults with ADHD may display higher dopamine transporter density. Meta-regression analyses showed density was higher in patients with previous medication exposure and lower in medication-naive patients.
Nakao	2011	Low	Adults with ADHD display (1) robustly localised global reductions in grey matter volume in the R lentiform nucleus (putamen & globus pallidus) extending to the caudate nucleus, and (2) slightly greater grey matter volume in the L posterior cingulate cortex.
Proal	2011	High	Mean cortical thickness was significantly reduced in adults diagnosed with childhood ADHD in comparison to controls, in the dorsal attentional network and limbic areas. Individuals diagnosed with childhood ADHD also displayed significantly decreased grey matter in the R caudate, R thalamus and bilateral cerebellar hemispheres, and cortical thinning in the bilateral parietal lobes, temporal poles, insula, precentral gyri, frontal poles, and R precuneus.
Ellison-Wright	2008	Low	Adults with ADHD displayed significant regional grey matter reduction the R putamen/globus pallidus region.
**Longitudinal Studies**			
Shaw	2013	High	Adult ADHD status is associated with altered development of components of the cortical networks that support attention, cognitive control, and the default mode network. Specifically, the R cingulate gyrus (extending to the R caudal regions and R isthmus, and extending to the R precuneus of the superior parietal lobe, R cuneus of the occipital lobe, and R fusiform gyrus), R dorso-lateral prefrontal cortex, L caudal anterior cingulate, L para-central lobule, L precuneus, and L postcentral gyrus.

Appendix I
Theme - ADHD and Atypical Brain Function

First Author	Year	Quality	Findings – In Comparison to Controls
Cui	2023	High	Adults with ADHD displayed reliable and robust age-related reduced fractional anisotropy in the splenium of the corpus collosum indicating white matter tract integrity reduces with increasing age.
Hou	2023	Mod	Children and adults with ADHD displayed smaller, weaker or inactive activation in the frontal cortex during working memory and inhibition control.
Liu	2023	Mod	Children with ADHD consistently exhibited hyper-connectivity between different parts of the cortex and L middle frontal gyrus, and hypo-connectivity between different parts of the cortex and L putamen/pallidus/amygdala. Adults with ADHD show hyper-connectivity between the cortex and R precuneus/sub-gyral/cingulate gyrus.
Zhang	2023	High	Children with ADHD do not display significant fractional anisotropy changes. Adolescents with ADHD displayed decreased fractional anisotropy in the corpus callosum (body and splenium), L optic radiations, R superior longitudinal fasciculus, L pons, inferior longitudinal fasciculus, and R corticospinal projections. Adults with ADHD displayed increased fractional anisotropy in the L superior longitudinal fasciculus, body of the corpus callosum and R precuneus as well as decrease FA clusters in the genu, body and splenium of the corpus callosum.
Connaughton	2022	Mod	Children and adolescents with ADHD tended to display widespread atypical white matter microstructure in both discrete white matter tracts and neural networks (mostly decreased connectivity but also increased in some areas). The most prominent differences were found in the fronto-striatal tracts, corpus callosum, superior longitudinal fasciculus, cingulum bundle, thalamic radiations, internal capsule and corona radiata.
Kowalczyk	2022	Mod	Across cognitive domains, children and adults with ADHD consistently displayed atypical functional connectivity in the cingulo-opercular, sensorimotor, visual, subcortical, and executive control networks, and similar sensorimotor and subcortical (primarily striatal) networks.
Michelini	2022	Mod	Children and adults with ADHD displayed broad brain-oscillatory alterations with small (theta) and small-to-moderate (alpha and beta) effect sizes.
Vidor	2022	High	Children with ADHD displayed higher concentrations of a composite of glutamate and glutamine in the R medial frontal area (both are thought to be implicated in neural pruning).
Zhao	2022	High	Children and adults with ADHD displayed decreased fractional anisotropy in the splenium of the corpus callosum indicating aberrant white matter microstructure.
Cortese	2021	Mod	Children and adults with ADHD displayed consistently dysregulated L superior temporal gyrus (hypo- and hyper-connectivity). Seed locations vary across individuals.
Yap	2021	Mod	Children and adults with ADHD displayed activation alterations in the frontal and parietal regions and poorer sustained attention and working memory, regardless of age and task paradigm along with large intra-subject variability.
Arora	2020	Low	The orbitofrontal and superior frontal cortices present with reduced activation and reduced cortical density in developmental ADHD. The dorsolateral prefrontal cortex has fractional anisotropy abnormalities and a reduced relative size.
Bellato	2020	Low	Although half the results were null, children and adults with ADHD tended to display atypical autonomic nervous system arousal, more often in the direction of hypo-arousal than hyper-arousal, particularly at rest and during tasks requiring response regulation and sustained attention. ANS function can be increased by stimulant medications, reinforcers and rewards.
Boon	2020	Low	Individuals with ADHD display white matter reductions and decreased prefrontal activity.
Kaiser	2020	Mod	Children and adults with ADHD displayed atypical event rate potential during inhibitory control, attention, working memory, and performance monitoring.

Lukito	2020	High	During cognitive control individuals with ADHD displayed under-activation in R thalamus/caudate, L middle temporal gyrus/superior temporal gyri/superior temporal pole, bilateral supplementary motor area/ dorsomedial prefrontal cortex, L inferior frontal gyrus/ anterior insula/ temporal pole, R anterior insula/putamen, L post-cingulate gyrus, L middle frontal gyrus/dorsolateral prefrontal cortices and R middle temporal gyrus. Age-stratified analyses: except for the L middle and superior temporal gyrus/temporal pole, (1) children displayed under-activated dorsomedial prefrontal cortex clusters and L post-central gyrus, and (2) adults displayed under-activation in all regions except the dorsomedial prefrontal cortex and L post-central gyrus. During motor response inhibition: under-activation in L middle frontal gyrus/dorsolateral prefrontal cortex, L anterior middle temporal gyrus/superior frontal gyrus, L post-central gyrus, R ventrolateral prefrontal cortex/orbital frontal cortex/anterior insula, R inferior frontal gyrus and R caudate. R Age-stratified analyses: (1) children displayed under-activation in L middle temporal gyrus/superior temporal gyrus, (2) adults displayed under-activation in L middle frontal gyrus/dorsolateral prefrontal cortex, (3) both age groups displayed under-activation in R ventrolateral prefrontal cortex /anterior insula.
Sutcubasi	2020	Mod	Children and adolescents with ADHD within-default mode network hypo- (i.e., posterior cingulate cortex seed) and hyper-connectivity (i.e., dorsal medial prefrontal cortex sub-system containing temporal pole-inferior frontal gyrus), and (2) within-cognitive control system hyperconnectivity. Children and adolescents also display reduced connectivity between the (3) default mode network and cognitive control and (4) affective/motivational and salience networks. Although less apparent in adulthood, these findings did not fully resolve.
Gao	2019	Mod	ADHD was characterised by hyperconnectivity between the frontoparietal network and regions of the default node network and affective network, as well as hypoconnectivity between the frontoparietal network and regions of the ventral attention network and somatosensory network.
Samae	2019	Mod	Children and adolescents with ADHD display variations of impaired communication and integration between the brain regions. During task-fMRI experiments using neutral stimuli, convergent aberrant findings were noted in the L pallidum/putamen. Decreased activity (in male subjects only) in the L inferior frontal gyrus was also found.
Aoki	2018	Mod	Children and adults with ADHD tended to display significantly lower fractional anisotropy values in four clusters: two in the corpus callosum (isthmus and posterior midbody), one in R inferior fronto-occipital fasciculus, and one in L inferior longitudinal fasciculus. Elevated fractional anisotropy in three clusters. The largest effect size was located in the L mid-cingulate, extending to the corpus callosum. The second largest cluster was situated in the anterior corpus callosum. The third cluster was in the L inferior fronto-occipital fasciculus. Results from different methodology did not converge.
Mauri	2018	Very Low	Children and adolescents with ADHD tended to show peculiar cortical activation both during neurological and emotional tasks, and lower (1) prefrontal cortex activation during tasks assessing attention, cognitive inhibition and flexibility, working memory and memory span, and (2) temporal cortex re-activity to facial emotional stimuli.
Mills	2018	High	Children with ADHD displayed reduced negative connectivity between task positive (i.e., fronto-parietal, cingulo-opercular and dorsal attention networks), and task negative networks (i.e., default mode network). Connectivity continues to become more negative between these networks throughout development.
Viera de Melo	2018	Low	Children with ADHD display (1) frontal lobe hypoactivation, specifically in the supplementary motor area, bilateral middle superior frontal gyrus, precentral gyrus and the inferior frontal gyrus, (2) parietal lobe hypoactivation in the frontal area and R postcentral gyrus, (3) parietal lobe hyperactivation in the R angular gyrus and R subparietal sulcus, (4) temporal lobe hypoactivation in the R superior temporal gyrus and in the occipital lobe, and (5) temporal lobe hyperactivation of the R middle occipital gyrus. These alternate patterns of activation were most frequently observed in the cingulate cortex (hypoactivation in the paracingulate gyrus, and hyperactivation in the R middle and posterior cingulate), insula (R sub-operculum white matter) and basal nuclei (hypoactivation in the putamen). Adults with ADHD display (1) frontal lobe hypoactivation in the precentral and R middle frontal gyri and central sulcus, and (2) parietal hyperactivation in the R angular gyrus.
Zhang	2018	High	Only males with ADHD displayed heterogenous significantly higher BDNF levels (a key molecule involved in differentiation of neuronal populations during development and plastic changes related to learning and memory).
Chen	2016	High	Individuals with ADHD display atypical fractional anisotropy in the commissural fibres that connect bilateral hemispheres, association fibres that link occipital, frontal and temporal regions. The most robust finds were located in the splenium of the corpus callosum that extended to the R cingulum, R sagittal stratum, and L tapetum. The projection fibres that pass through the corona radiata and cerebellum may also be affected although sensitivity analysis failed to retain significance.
Cheng	2016	High	Compared to the typically developed children, individuals with ADHD demonstrated reduced mismatched negativity amplitudes.
Cortese	2016	Low	Adults with ADHD displayed hypoactivation in the L putamen, L inferior frontal gyrus (pars opercularis), L temporal pole, and R caudate.
Dutra	2016	Mod	Children and adults with ADHD displayed short intracortical inhibition. No differences in resting motor threshold and silent period.

Lei	2015	Mod	Individuals with ADHD displayed significantly decreased activation during response inhibition in the supplementary motor area, insula, caudate, and precentral gyrus, as well as increased activation in the postcentral gyrus, inferior frontal gyrus, and precuneus.
McCarthy	2014	Mod	Across all tasks, individuals with ADHD displayed significantly less frontal lobe activity N-back task: less activity in the bilateral superior frontal gyri and L medial frontal gyrus. Go/no-go tasks: less mean activation in the L medial frontal gyrus and R caudate. Stop tasks: children displayed less activity the bilateral inferior frontal gyri, R superior frontal gyrus and R middle frontal gyri.
Posner	2014	Very Low	ADHD is associated with heterogenous anticorrelations (1) between the cognitive control network and the default mode network which may result in mind wandering that impedes sustained attention, (2) within the default node network, (3) within the cortioco-striato-thalamo-cortical loops (cognitive and limbic).
Aoki	2013	High	Childhood ADHD is associated with significantly higher-than-normal N-acetylaspartate, a marker of neuronal activity, in the medial prefrontal cortex.
Arns	2013	Low	Children and adolescents with ADHD display differences in Theta/Beta ratio (moderate effect size). Results were marred by significant heterogeneity and are likely to be an over estimation. Post hoc tests revealed a strong relationship between effect size and year of publication.
Hart	2013	Low	Individuals with ADHD display reduced activation in the brain regions associated with (1) response inhibition (the fronto-basal ganglia-thalamic networks involving the R inferior frontal cortex, supplementary motor area, and anterior caudate, and (2) attentional regulation (dorsolateral prefrontal cortex, parietal, and cerebellar). Age effects could be analysed only for the inhibition meta-analysis, showing that the supplementary motor area and basal ganglia were under activated solely in children with ADHD, while the inferior frontal cortex and thalamus were under activated solely in adults with ADHD.
Karalunas	2013	Low	Children with ADHD demonstrated more low-frequency reaction variability as well as equivalent excess reaction variability in a faster frequency comparison band.
Shaw	2013	High	Adult ADHD status is associated with altered development of components of the cortical networks that support attention, cognitive control, and the default mode network, Specifically, the R cingulate gyrus (extending to the R caudal regions and R isthmus, and extending to the R precuneus of the superior parietal lobe, R cuneus of the occipital lobe, and R fusiform gyrus), R dorso-lateral prefrontal cortex, L caudal anterior cingulate, L para-central lobule, L precuneus, and L postcentral gyrus.
Cortese	2012	High	Children with ADHD displayed significant (1) hypoactivation in the frontal regions and putamen bilaterally, and in R parietal and R temporal regions, and (2) hyperactivation in the R angular gyrus, middle occipital gyrus, posterior cingulate cortex, and midcingulate cortex. From a network perspective, children with ADHD display hypoactivation in the frontoparietal control network and ventral attentional network, and significant (2) hyperactivation in the default, ventral attention, and somatomotor networks. Stimulant-naïve children only displayed hypoactivation in the R superior temporal gyrus, bilateral putamen and R thalamus. Adults with ADHD displayed: (1) hypoactivation in the R central sulcus, precentral gyrus, and middle frontal gyrus, and frontoparietal system; and (2) hyperactivation in a region with a peak in the R angular and middle occipital gyri, and in the visual, dorsal attention, and default networks.
Hart	2012	Mod	Individuals with ADHD displayed reduced activation in typical areas related to timing such as L inferior prefrontal cortex, insula, cerebellum, and L inferior parietal lobe. They also consistently displayed enhanced activation relative to controls in precuneus and posterior cingulate, presumably reflecting problems with deactivation of the default mode network.
Rash	2012	Low	Findings were not unanimous but suggested that children with unmedicated ADHD experienced lower levels of cardiac vagal tone than healthy controls.
Van Ewijk	2012	Mod	Children and adults with ADHD display wide spread alterations in white matter integrity, most evidently in the R anterior corona radiata, R forceps minor, bilateral internal capsule, and L cerebellum. Other areas that were less reliably implicated include the inferior and superior longitudinal fasciculus, cingulum, corpus collosum, caudate, basal ganglia, uncinate fasciculus, forceps minor, frontal, temporal, parietal and occipital lobes.
Proal	2011	High	Cortex of individuals with ADHD were significantly thinner in the dorsal attentional network and limbic areas. Additionally, grey matter was significantly decreased in right caudate, right thalamus and bilateral cerebellar hemispheres.
Szuromi	2011	Low	Adults with ADHD display significant medium size affect reduced P3 amplitude in comparison to controls.
Paloyelis	2007	Very Low	Individuals with ADHD display both lower and higher brain activity in different regions of the brain dependent on task.
Dickstein	2006	Low	Across studies, children and adults with ADHD displayed hypoactivity within the fronto-striatal and fronto-parietal circuits. The areas affected included the anterior cingulate, dorsolateral prefrontal, and inferior prefrontal cortices, as well as related regions including basal ganglia, thalamus, and portions of parietal cortex.
Snyder	2006	Mod	Individuals with ADHD displayed an increased theta/beta ratio.

Appendix J
Sub-Theme: ADHD and Atypical Working Memory

First Author	Year	Quality	Findings – In Comparison to Controls
Hou	2023	Mod	Children and adults with ADHD displayed smaller, weaker or inactive activation in the frontal cortex during working memory tasks.
Michellini	2022	Mod	Children and adults with ADHD displayed broad brain-oscillatory alterations with small (theta) and small-to-moderate (alpha and beta) effect sizes during working memory tasks.
Orban	2022	High	Individuals with ADHD display worse control over memory and differences in verbal memory interference control, and executive control process involved in working memory. Effects were different depending upon interference type and participant age.
Loyer-Carbonneau	2021	High	Boys and girls with ADHD presented with significantly more working memory deficits.
Onandia-Hinchado	2021	Mod	Adults with ADHD display a cognitive profile characterised by cognitive deficits including atypical working memory.
Yap	2021	Mod	Individuals with ADHD displayed poorer working memory.
Boon	2020	Low	Stroop task studies have shown that adults with ADHD combined subtype have a reduced activation in areas of the prefrontal cortex which correlated with measures of working memory.
Kaiser	2020	Mod	Children and adults with ADHD displayed on average atypical event rate potential during working memory tasks.
Ramos	2020	Mod	Children and adolescents with ADHD exhibit poorer working memory performance during digital scan backwards performance, with a medium-sized effect.
LeRoy	2019	Low	Individuals with ADHD-C and ADHD-I appear to perform worse on measures of working memory.
Mauri	2018	Very Low	The majority of the considered studies found lower activations in prefrontal cortices of children and adolescents with ADHD compared to controls during tasks assessing working memory and memory span.
Pievsky	2018	Mod	ADHD is associated with substantial heterogenous moderate to large deficits across a variety of neurocognitive domains including working memory.
Cheng	2016	High	Children and adolescents with ADHD demonstrated reduced miss-matched negativity amplitudes, indicating poor auditory sensory memory (working memory).
McCarthy	2014	Mod	Across all tasks, in comparison to controls, adolescents and adults with ADHD displayed significantly less frontal lobe activity compared to controls. Less exposure to treatment and lengthier wash-out times resulted in less L medial frontal cortex activation in N-back and Go/no-go studies. The N-back task measures the neural components underlying working memory performance.
Alderson	2013	Mod	Adults with ADHD display moderate heterogenous phonological and visuospatial working memory deficits relative to controls.
Cortese	2012	High	Children with ADHD displayed hypoactivation during working memory tasks in the L inferior frontal gyrus, anterior insula and R middle frontal gyrus.
Kasper	2012	Mod	Children with ADHD exhibit heterogenous, statistically significant, large magnitude phonological and visuospatial working memory task deficits.
Paloyelis	2007	Very Low	In analyses that examined working memory children and adults with ADHD displayed lower brain activity.
Boonstra	2005	Low	Adults with ADHD displayed atypical verbal fluency.
Schoechlin	2005	Mod	Adults with ADHD scored lower in the domains of working memory.
Wilcutt	2005	Mod	ADHD is associated with weaknesses in several key executive function domains including working memory.
Hervey	2004	Low	Adults with ADHD displayed atypical neuropsychological working memory function.

Appendix K
Sub-Theme: ADHD and Atypical Inhibitory Control (Response Inhibition and Interference Control)

Note: During neuropsychology studies:

- Atypical response inhibition is indexed by excessive body movement, rapid responses, delay aversion, choice impulsivity, sacrificing accuracy for speed, diminished post-error slowing, omission errors, commission errors, problems delaying gratification, and risky decision-making.
- Atypical interference control (required for attention regulation) is indexed by reaction time (delay and variability), vigilance (continuous performance (mind wandering), commission errors), and sustained attention.

First Author	Year	Quality	Findings – In Comparison to Controls
Hou	2023	Mod	Children and adults with ADHD displayed smaller, weaker or inactive activation in the frontal cortex during inhibition control.
Chamorro	2022	High	Individuals with ADHD commit more ocular inhibition errors in the form of anticipatory rapid eye movement responses during memory guided saccade task waiting periods. This suggests that the capacity to withhold a response on presentation of a relevant stimulus in impaired in adolescents and adults with ADHD.
Michelini	2022	Mod	Children and adults with ADHD displayed broad brain-oscillatory alterations with small (theta) and small-to-moderate (alpha and beta) effect sizes during cognitive control and simple attention tasks.
Orban	2022	High	Individuals with ADHD display differences in verbal memory interference control. Effects were different depending upon interference type and participant age.
Senkowski	2020	High	Adults with ADHD display reliable inhibitory control deficits, as expressed in prolonged stop signal task response times, with a moderate effect size.
Loyer-Carbonneau	2021	High	Boys and girls with ADHD presented significant motor response inhibition and interference control deficits. Boys with ADHD tend to display more hyperactivity as well as more difficulties with response inhibition in comparison to girls with ADHD.
Onandia-Hinchado	2021	Mod	Adults with ADHD displayed atypical inhibition with an emphasis on reward delay, interference control and commission errors.
Lanier	2021	Low	ADHD is frequently associated with impairing spontaneous mind wandering.
Marx	2021	Mod	Individuals with ADHD tend to choose small immediate rewards over large delayed rewards more frequently than controls (make impulsive choices).
Roberts	2021	Mod	Children and adults with ADHD exhibit moderately more risk-taking compared to children and adults without the disorder.
Valmiki	2021	Mod	Male adolescents with ADHD are more responsive to reinforcement related to monetary gain and loss and are more sensitive to larger rewards than smaller ones.
Boon	2020	Low	ADHD is associated with reduced basal ganglia volumes (which has been linked to poor impulse control). Reduced basal ganglia, thalamus, cerebellum volumes, grey matter, which has been linked to poor impulse control, attention regulation and alerting attention.
Bellato	2020	Low	Children and adults tend to display autonomic nervous system hyperarousal during cognitive tasks requiring response regulation.
Kaiser	2020	Mod	Children and adults with ADHD displayed on average atypical event rate potential during inhibitory control, and performance monitoring. On average individuals with ADHD display on average smaller Cue-P300-amplitudes, longer Go-P300-latencies, smaller NoGo-P300-amplitudes, longer NoGo-P300-latencies, smaller CNV-amplitudes, and smaller Pe-amplitudes. These findings have also been linked to poor attentional selective attention i.e., stimulus expectation, resource association and attention reorientation, motor and non-motor pre and ongoing processing after cue stimulus.
Lukito	2020	High	ADHD is associated with under activation in a number of brain regions during cognitive control and motor response inhibition including the R inferior frontal gyrus and caudate.

Sutcubasi	2020	Mod	ADHD is associated with (1) reduced resting state connectivity between default mode network hubs, which has been linked to excessing mind wandering and attentional difficulties during task performance, and (2) reduced connectivity between default mode network seeds and the central control network, which has been linked to lapses in attention.
Gao	2019	Mod	Children and adults with ADHD displayed (1) hypoconnectivity between the frontoparietal network and the precentral gyrus, which is involved in response inhibition, and (2) hyperconnectivity between the affective network and pre-frontal regions of the default mode network and the frontoparietal network, which has been linked to greater impulsivity.
LeRoy	2019	Low	Individuals with ADHD-C and ADHD-I appear to perform worse on measures of executive functioning (including tests measuring cognitive inhibition), and measures of attention (tests measured orientating attention, response time, vigilance).
Patros	2019	High	Children with ADHD responded more quickly on planning tasks when compared with typically developing peers.
Samea	2019	Mod	Aberrant finding in the task-fMRI experiments (using neutral stimuli) in the L pallidum/putamen. Aberrant ventral putamen, medial orbitofrontal cortex and inferior frontal gyrus activation has been linked to impulsivity, disinhibition and hyperactivity.
Mauri	2018	Very Low	The majority of the considered studies found lower activations in prefrontal cortices of children with ADHD compared to controls during tasks assessing cognitive inhibition and attention abilities.
Mills	2018	High	Children with ADHD displayed poorer signal detection on the continuous performance task (reduced attentional vigilance), more so on easy than difficult tasks.
Pievsky	2018	Mod	ADHD is associated with substantial heterogenous moderate to large deficits across a variety of neurocognitive domains including response inhibition.
Viera de Melo	2018	Low	Children and adults with ADHD display regional specific hypo- and hyperactivation patterns. A hypoactivation pattern along the ventral attention network and a partial hypoactivation along the dorsal attention network has been linked to poor inhibition.
Balogh	2016	Mod	Children and adolescents with ADHD displayed significantly diminished post-error slowing in compared to controls. Post error individuals with ADHD participants sustained or even increased their speed compared to controls who slow down post error.
Ma	2016	Low	Reinforcement may ameliorate response inhibition in individuals with ADHD more than in controls and may normalise performance to the baseline level of controls.
Patros	2016	High	Children and adolescents with ADHD exhibited moderately increased impulsive decision-making compared to controls during both delay of gratification and delay discounting tasks.
Dekkers	2016	High	ADHD is related to increased risky decision making on gambling tasks in laboratory settings.
Dutra	2016	Mod	Individuals with ADHD displayed short intracortical inhibition (a marker of motor inhibition). Although the magnitude of the behaviour inhibition challenges children and adolescents with ADHD display appear to decrease with age, the majority of individuals with ADHD continue to display poor response inhibition in adulthood.
Jackson	2016	Mod	Individuals with ADHD, significantly elevated robust problems with delay discounting.
Lei	2015	Mod	Individuals with ADHD displayed significantly decreased activation during response inhibition in the supplementary motor area, insula, caudate, and precentral gyrus, as well as increased activation in the postcentral gyrus, inferior frontal gyrus, and precuneus. The activation decreases in the R caudate were greater in child ADHD patients than adult ADHD patients.
Mowinckel	2015	Mod	Individuals with ADHD display attention deficits, measured with the continuous performance tasks, commission errors, omission errors, and reaction time variance.
Pauli-Pott	2015	Mod	Individuals with ADHD display differences in inhibitory control and delay aversion/discounting. These differences reduce with age.
Karalunas	2014	Mod	Children and young adults with ADHD display reaction time variability in comparison to healthy controls and children with ASD alone.

McCarthy	2014	Mod	Across all tasks, in comparison to controls, individuals with ADHD displayed significantly less frontal lobe activity compared to controls during N-back and Go/No-go studies. The N-back task measures the neural components underlying working memory performance. Working memory accuracy necessitates the inhibition of responses to irrelevant task stimuli. The Go/no-go task measures selective motor response inhibition.
Posner	2014	Very Low	ADHD is associated with heterogenous anticorrelations between cortioco-striato-thalamo-cortical loops (cognitive and limbic). Atypical connectivity in both the cognitive cortioco-striato-thalamo-cortical loops in ADHD has been linked to atypical inhibition, while anti-correlations between the cognitive control network and the default mode network may result in mind wandering that impedes sustained attention.
Groen	2013	Low	Individuals with ADHD display increased risky performance on gambling tasks is mixed. The findings are stronger for children/adolescents with ADHD than for adults with ADHD.
Geburek	2013	Low	Adolescents and adults with ADHD responded slower during flanker tasks and made more errors during GoNogo and flanker tasks, indicating a response deficit is a central feature of the ADHD syndrome.
Hart	2013	Low	Children and adults with ADHD displayed reduced activation for (1) inhibition in the R inferior frontal cortex, supplementary motor area, and anterior cingulate cortex, as well as striato-thalamic areas, and (2) attention (interference control) in the R dorso-lateral prefrontal cortex, posterior basal ganglia, and thalamic and parietal regions. Inhibition meta-analysis showed the supplementary motor area and basal ganglia were under activated solely in children with ADHD, while the inferior frontal cortex and thalamus were under activated solely in adults with ADHD.
Karalunas	2013	Low	Children with ADHD demonstrate more low-frequency reaction time variability than non-ADHD controls as well as equivalent excess reaction variability in a faster frequency comparison band.
Kofler	2013	High	Children and adults with ADHD demonstrated impairments in reaction time variability relative to controls.
Shaw	2013	High	Adult ADHD status is associated with altered development of components of the cortical networks that support cognitive control.
Cortese	2012	High	Children with ADHD displayed hypoactivation during inhibition tasks in several frontal regions bilaterally, and the R superior temporal gyrus, R thalamus and midbrain.
Huang-Pollock	2012	Mod	School aged children with ADHD display a large effect size for overall performance on tasks of sustained attention. Effect size was large for commission errors.
Metin	2012	Low	During trials with slow event rates, children and adults with ADHD displayed significant and disproportionate slowing of reaction time relative to controls. During trials examining the influence of fast event rates on commission errors, individuals with ADHD displayed disproportionally more commission errors than controls.
Rash	2012	Low	Findings were not unanimous but suggested that children with unmedicated ADHD experienced lower levels of cardiac vagal tone than controls during tasks that involved self-regulation and emotion regulation.
Huizenga	2009	Mod	Inhibitory dysfunction in children and adolescents with ADHD Is moderated by task complexity and is most pronounced for spatially noncompatible responses.
Mullane	2009	Low	Children with ADHD display poorer interference control as indexed by reaction time, significantly larger accuracy congruency effects, and significantly larger inverse efficiency congruency effects.
Lansbergen	2007	Low	Interference control is consistently compromised in individuals with ADHD.
Paloyelis	2007	Very Low	In analyses that examined inhibition errors children and adults with ADHD displayed lower brain activity.
Dickstein	2006	Low	Children and adults with ADHD displayed hypoactivation in the inferior prefrontal cortex, medial wall regions, and the precentral gyrus during response inhibition tasks.
Boonstra	2005	Low	Adults with ADHD displayed atypical inhibition.
Schoechlin	2005	Mod	Adults with ADHD display problems with simple attention, sustained attention, focused attention.
Willcutt	2005	Mod	ADHD is associated with weaknesses in several key executive function domains including inhibitory control.

Hervey	2004	Low	Neuropsychological deficits were found to be expressed in adults with ADHD across multiple domains of functioning, with notable impairments in response inhibition and distractibility.
Oosterlaan	1998	Low	Children with ADHD display poor response inhibition related to a slow inhibitory process, as do children with conduct disorder, although the findings were inconsistent for conduct disorder children. Children with ADHD and conduct disorder display greater impairments than children with only ADHD.

Appendix L
Sub-Theme: ADHD and Atypical Cognitive Flexibility

First Author	Year	Quality	Findings – In Comparison to Controls
Loyer-Carbonneau	2021	High	Children with ADHD display problems with cognitive flexibility. Boys with ADHD tend to display more difficulties than girls.
Boon	2020	Low	During task switching, deficits in activation of the basal ganglia and cingulate gyri, and prefrontal activity were found in children and adults.
Sutcubasi	2020	Mod	Children and adults displayed within-default mode network hypo- and hyper-connectivity, and within-cognitive control system hyperconnectivity. Reduced resting state connectivity between default mode network hubs has been linked to impaired inter-temporal decision-making.
Gao	2019	Mod	Children and adults with ADHD displayed hypoconnectivity between the frontoparietal network and the precentral gyrus, which has been linked to problems with task switching.
LeRoy	2019	Low	Individuals with ADHD-C and ADHD-I appear to perform worse on measures of executive functioning including tests measuring cognitive flexibility.
Mauri	2018	Very Low	Children and adolescents with ADHD display lower prefrontal cortex activation during tasks assessing cognitive flexibility.
Pievsky	2018	Mod	ADHD is associated with substantial heterogenous moderate to large deficits across a variety of neurocognitive domains including set shifting.
Boonstra	2005	Low	Adults with ADHD display atypical set-shifting.
Schoechlin	2005	Mod	Adults with ADHD displayed problems with cognitive fluency.

Appendix M
Sub-Theme: ADHD and Atypical Alerting Attention and Vigilance

Note: Alerting attention is the arousal component of attention regulation. It encompasses maintaining attentional alertness (arousal) and sustaining vigilance. Vigilance involves the ability to maintain a tonic state of alertness and wakefulness during prolonged and sustained mental activity (effort), and the ability to detect a rare critical event (Huang-Pollock & Nigg, 2003). Measures: sustained attention to a response task, omission errors, error monitoring, global reaction times. The ventral attention network tends to typically respond when behaviourally relevant stimuli occur unexpectedly (Vossel et al., 2014).

First Author	Year	Quality	Findings – In Comparison to Controls
Loyer Carbonneau	2021	High	Boys and girls with ADHD presented significantly more sustained attention deficits.
Onandia-Hinchado	2021	Mod	Adults with ADHD display a cognitive profile characterised by cognitive deficits across all attention modalities i.e., (alertness, selective and sustained attention).
Yap	2021	Mod	Individuals with ADHD displayed poorer sustained attention.
Arora	2020	Low	Children with ADHD displayed differences in alerting attention network function.
Bellato	2020	Low	Children and adults with ADHD tended to display atypical autonomic nervous system arousal, more often in the direction of hypo-arousal than hyper-arousal, particularly during tasks requiring sustained attention.
Kaiser	2020	Mod	Children and adults with ADHD displayed on average atypical event rate potential during inhibitory control, and performance monitoring. On average individuals with ADHD display on average smaller Cue-P300-amplitudes, longer Go-P300-latencies, smaller NoGo-P300-amplitudes, longer NoGo-P300-latencies, smaller CNV-amplitudes, and smaller Pe-amplitudes. These findings have also been linked to poor attentional selective attention i.e., stimulus expectation, resource association and attention reorientation, motor and non-motor pre and ongoing processing after cue stimulus.
Senkowski	2020	High	Adults with ADHD may display greater stop signal task omission errors and reduced go accuracy, indicative of altered sustained attention.
Sutcubasi	2020	Mod	ADHD is associated with (1) reduced resting state connectivity between default mode network hubs, which has been linked to attentional difficulties during task performance, and (2) reduced connectivity between default mode network seeds and the central control network, which has been linked to lapses in attention.
Gao	2019	Mod	ADHD is characterised by hypoconnectivity between the regions of the ventral attention network.
LeRoy	2019	Low	Individuals with ADHD-C and ADHD-I performed worse on measures of attentional vigilance and response time.
Mauri	2018	Very Low	The majority of the considered studies found lower activations in prefrontal cortices of children with ADHD compared to controls during tasks assessing attention abilities.
Mills	2018	High	Children with ADHD displayed poorer signal detection on the continuous performance task (reduced attentional vigilance), more so on easy than difficult tasks.
Pievsky	2018	Mod	ADHD is associated with substantial heterogenous moderate to large deficits across a variety of neurocognitive domains including vigilance.
Cheng	2016	High	Children and adolescents with ADHD demonstrated a reduced mismatched negativity amplitude compared to healthy controls. Mismatched negativity is elicited by any discernible deviant sound. Individuals with ADHD demonstrated reduced mismatched negativity amplitudes, indicating less vigilance to auditory change and reduced involuntary attention switching.
Geburek	2013	Low	Adolescents and adults with ADHD responded slower during flanker tasks and made more errors during GoNogo and flanker tasks, indicating a monitoring deficit is a central feature of the ADHD syndrome.

Shaw	2013	High	Adult ADHD status is associated with altered development of components of the cortical networks that support attention, including the cingulate gyrus and medial prefrontal cortex, extending to the precuneus and the R dorsolateral prefrontal cortex. The cingulate is a key hub of the cingulo-operculum network that monitors for behaviourally salient stimuli and maintains task performance.
Cortese	2012	High	During vigilance or attention tasks, children with ADHD displayed significant hypoactivation and hyperactivation only in the R paracingulate gyrus in the ventral attention network.
Huang-Pollock	2012	Mod	School aged children with ADHD displayed greater perceptual sensitivity (difficulty distinguishing targets from non-targets) and slower drift rates during tasks requiring sustained attention. They may also display more omission errors.
Szuromi	2011	Low	Adults with ADHD displayed significant medium size affect reduced P3 amplitude in comparison to controls. Target-related P3 is thought to reflect the activation of cortical areas, particularly that of the temporoparietal junction, which can be associated with the functioning of the ventral attention network which is involved in detecting stimulus (stimulus driven attentional control).
Mullane	2008	Low	Children with ADHD demonstrated less efficient serial search. These findings indicate children with ADHD show impairments in aspects of effortful visual selective attention, as measured by visual search.
Alderson	2007	Mod	Children with ADHD displayed significantly slower mean reaction time, greater reaction time variability, and slower stop-signal reaction time. These differences appear to reflect a generalised problems with attention/cognitive processing rather than behavioural inhibition.
Cortese	2006	Mod	Children with ADHD fall asleep more often on multiple sleep latency testing and may display excessive daytime sleepiness which impacts alertness.
Willcutt	2005	Mod	ADHD is associated with weaknesses in several key executive function domains including attention vigilance.

Appendix N
Sub-Theme: ADHD and Atypical Reward Processing

Atypical reward processing and motivation is indexed by delay aversion, preferences for immediate rewards, a heightened sensitivity to reward, and increased temporal discounting.			
**First Author**	**Year**	**Quality**	**Findings – In Comparison to Controls**
Doidge	2021	Mod	During temporal discounting and delay of gratification tasks, females with ADHD chose smaller immediate rewards over larger delayed rewards more often than males with ADHD. In contrast, male with ADHD chose were more likely to choose the larger delayed reward.
Onandia-Hinchado	2021	Mod	Adults with ADHD display a cognitive profile characterised by cognitive deficits including reward delay.
Marx	2021	Mod	Individuals with ADHD tend to choose small immediate rewards over large delayed rewards more frequently than controls (make impulsive choices).
Boon	2020	Low	Individuals with ADHD display abnormal activity in the basal ganglia during reward anticipation.
Bellato	2020	Low	Children and adults with ADHD have a tendency to display autonomic nervous system hypoarousal during reward tasks. Rewards and reinforcers provided to individuals with ADHD may help them to up-regulate their attentional and arousal state.
Lukito	2020	High	Under-activation in R inferior frontal gyrus and caudate has been linked to atypical reward-based decision-making.
Sutcubasi	2020	Mod	Children and adults displayed within-default mode network hypo- and hyper-connectivity, and within-cognitive control system hyperconnectivity. Hyper-activation in default mode network regions during information processing, which has been linked to disrupted cognitive performance when participating in goal related tasks.
Jackson	2016	Mod	Children and adults with ADHD significantly elevated problems with delay discounting, which reflects how much a person devalues a reward based on its delay in time.
Ma	2016	Low	Reinforcement may ameliorate response inhibition problems in children and adolescents with ADHD more than in controls and may normalise performance to the baseline level of controls (motivation).
Patros	2016	High	Children and adolescents with ADHD exhibited moderately increased impulsive decision-making compared to controls during both delay of gratification and delay discounting tasks.
Pauli-Pott	2015	Mod	Children and adults with ADHD display differences in inhibitory control and delay aversion/discounting. These differences change with age.
Plichta	2014	Very low	Individuals with ADHD displayed ventral-striatal hypo-responsiveness during task measuring reward anticipation.
Groen	2013	Low	Children and adults with ADHD often displayed increased risky performance during gambling tasks, although results are highly heterogenous. The risk is stronger for children/adolescents with ADHD than for adults with ADHD. Comorbid ODD/CD appears to increase the risk.

Appendix O
Sub-Theme: ADHD and Atypical Long-Term Memory

First Author	Year	Quality	Findings – In Comparison to Controls
Orban	2022	High	Adults and children with ADHD exhibited more retroactive verbal memory interference control problems and performed worse on memory control tasks.
Hulsbosch	2021	High	Although the evidence is limited, children with ADHD did not display clear problems with basic instrumental learning under laboratory conditions, but may display problems when task complexity increases. Children displayed problems with conditional discrimination learning, a complex form of learning, particularly when there is a delay between the discriminative cue and the opportunity to respond. It is possible that they also display difficulties with reversal learning.
Boon	2020	Low	The grey matter abnormalities displayed by children with ADHD have been linked to atypical memory.
LeRoy	2019	Low	Adults with ADHD-C and ADHD-I appear to perform worse on measures of memory.
Talbot	2018	Low	Children with ADHD perform significantly worse on time-based prospective and event-based prospective memory tasks.
Hoogman	2017	High	Children with ADHD displayed reduced hippocampus volumes. The hippocampus plays a major role in learning and memory.
Skodzik	2017	Mod	Adults with ADHD performed significantly worse than controls on verbal but not on visual long-term memory and memory acquisition subtests. Memory deficits in adult with ADHD may reflect a learning deficit induced at the stage of encoding.
Sanjeevan	2020	High	Procedural sequence learning appears to be preserved in ADHD.
Boonstra	2005	Low	Adults with ADHD displayed atypical verbal fluency.
Schoechlin	2005	Mod	Adults with ADHD display problems with verbal and figural (visual) memory.
Hervey	2004	Low	Adults with ADHD display notable verbal long-term memory deficits, generally revolved around memory retrieval and encoding, and the ability to recognise and organise words via semantic clustering.

Appendix P
Sub-Theme: ADHD and Atypical Reaction Time and Processing Speed

First Author	Year	Quality	Findings – In Comparison to Controls
Onandia-Hinchado	2021	Mod	Adults with ADHD display a cognitive profile characterised by cognitive deficits including slower processing speed and slower performance for divided attention in terms of managing multiple simultaneous sources of information.
Dan	2020	Very Low	Adolescents with ADHD display no differences in reaction time, reaction time variance and cognition accuracy when recognising emotional facial expressions, however, brain activity and temporal evolution differences are present when neural responses are recorded using f-MRI or event related potentials.
LeRoy	2019	Low	Individuals with ADHD-C and ADHD-I appear to perform worse on measures processing speed.
Pievsky	2018	Mod	ADHD is associated with substantial heterogenous moderate to large deficits across a variety of neurocognitive domains including atypical reaction time.
Karalunas	2014	Mod	Children and young adults with ADHD display reaction time variability.
Karalunas	2013	Low	Children with ADHD demonstrated more low-frequency reaction variability as well as equivalent excess reaction variability in a faster frequency comparison band.
Kofler	2013	High	Children with ADHD demonstrated robust but heterogenous large magnitude impairments in reaction time variability relative to controls. Adults with ADHD continue to demonstrate moderate magnitude increased reaction time variability. Processing speed was unaffected when reaction time variability is accounted for.
Huang-Pollock	2012	Mod	Children with ADHD committed more errors and had slower/more variable reaction times than non-ADHD controls.
Metin	2012	Low	Children and adults with ADHD displayed significant and disproportionate slowing of reaction time on trials with slow event rates.
Huizenga	2009	Mod	Inhibitory dysfunction in children and adolescents with ADHD, assessed by the stop-signal reaction time, is moderated by task complexity. Results differed significantly according to task complexity (they increased with increased with task complexity).
Alderson	2007	Mod	Children with ADHD displayed heterogenous significantly slower mean reaction time, greater reaction time variability, and slower stop-signal reaction time. These differences appear to reflect generalised problems with attention/cognitive processing rather than behavioural inhibition.
Boonstra	2005	Low	Adults with ADHD display atypical reaction times.
Lijffijt	2005	Low	Children with ADHD but not adults with ADHD display significantly longer basic reaction time. Adults with ADHD display a slowing of stop sign reaction time relative to mean reaction time.

Appendix Q
Sub-Theme: ADHD and Atypical Time Perception, Planning and Complex Decision Making/Problem Solving

First Author	Year	Quality	Findings – In Comparison to Controls
Mette	2023	Low	Adults with ADHD may display a deficit in time estimation, time reproduction and time management although results are equivocal.
Zheng	2022	High	Children/adolescents with ADHD perceived time less accurately and less precisely and displayed a higher tendency to overestimate time than controls.
Loyer-Carbonneau	2021	High	Boys and girls with ADHD presented significantly more planning deficits.
Boon	2020	Low	Individuals with ADHD display reduced (1) basal ganglia volumes, which has been linked to atypical planning, prioritising and reasoning, (2) activity in regions associated with sensorimotor timing which is needed for accurate responses to these tasks.
LeRoy	2019	Low	Individuals with ADHD-C and ADHD-I appear to perform worse on measures of cognitive planning.
Nejati	2020	High	Children with ADHD displayed significant difficulties in time perception. The problem is obvious in different types of modalities including visual and auditory, in different types of task time estimation, and during time reproduction and anticipation, and especially in longer duration.
Patros	2019	High	Children with ADHD experience moderate-magnitude planning deficits.
Pievsky	2018	Mod	ADHD is associated with substantial heterogenous moderate to large deficits across a variety of neurocognitive domains including atypical planning/organisation.
Dekkers	2016	High	ADHD is related to increased risky decision making on gambling tasks in laboratory settings.
Patros	2016	High	Children and adolescents with ADHD exhibited moderately increased impulsive decision-making compared to controls during both delay of gratification and delay discounting tasks.
Mowinckel	2015	Mod	Adults with ADHD display decision-making deficits which are of similar magnitude to attention deficits.
Hart	2012	Mod	Individuals with ADHD displayed reduced activation in typical areas related to timing.
Schoechlin	2005	Mod	Adults with ADHD display problems with visual/figural problem-solving, abstract problem-solving and verbal intelligence (reasoning).
Willcutt	2005	Mod	ADHD is associated with weaknesses in several key executive function domains including planning.
